# Gut Microbiota Linked to Sexual Preference and HIV Infection

**DOI:** 10.1016/j.ebiom.2016.01.032

**Published:** 2016-01-28

**Authors:** Marc Noguera-Julian, Muntsa Rocafort, Yolanda Guillén, Javier Rivera, Maria Casadellà, Piotr Nowak, Falk Hildebrand, Georg Zeller, Mariona Parera, Rocío Bellido, Cristina Rodríguez, Jorge Carrillo, Beatriz Mothe, Josep Coll, Isabel Bravo, Carla Estany, Cristina Herrero, Jorge Saz, Guillem Sirera, Ariadna Torrela, Jordi Navarro, Manel Crespo, Christian Brander, Eugènia Negredo, Julià Blanco, Francisco Guarner, Maria Luz Calle, Peer Bork, Anders Sönnerborg, Bonaventura Clotet, Roger Paredes

**Affiliations:** a IrsiCaixa AIDS Research Institute, Ctra de Canyet s/n, 08916 Badalona, Catalonia, Spain; b Universitat de Vic-Universitat Central de Catalunya, C. Sagrada Família 7, 08500 Vic, Catalonia, Spain; c Universitat Autònoma de Barcelona, 08193 Bellaterra, Catalonia, Spain; d Department of Medicine, Unit of Infectious Diseases, Karolinska University Hospital, Karolinska Institutet, Huddinge 141, 86, Stockholm, Sweden; e Structural and Computational Biology, European Molecular Biology Laboratory, Meyerhofstrasse 1, 69117 Heidelberg, Germany; f HIV Unit & Lluita Contra la SIDA Foundation, Hospital Universitari Germans Trias i Pujol, Ctra de Canyet s/n, 08916 Badalona, Catalonia, Spain; g ISGLOBAL, Carrer Rosselló, 132, 08036 Barcelona, Catalonia, Spain; h BCN Checkpoint, Carrer del Comte Borrell, 164, 08015 Barcelona, Catalonia, Spain; i Infectious Diseases Unit, Hospital Universitari Vall d'Hebrón, Passeig de la Vall d'Hebrón, 119–129, 08035 Barcelona, Catalonia, Spain; j Institució Catalana de Recerca i Estudis Avançats (ICREA), Barcelona, Catalonia, Spain; k Digestive Diseases Department, Vall d'Hebrón Institute of Research, Hospital Universitari Vall d'Hebrón, Passeig de la Vall d'Hebrón, 119–129, 08035 Barcelona, Catalonia, Spain; l Max-Delbrück-Centre for Molecular Medicine, Robert-Rössle-Str. 10, 13092 Berlin, Germany; m Molecular Medicine Partnership Unit, EMBL, Meyerhofstrasse 1, 69117 Heidelberg, Germany

**Keywords:** HIV-1, Microbiome, Microbiota, 16S rDNA, *Prevotella*, *Bacteroides*

## Abstract

The precise effects of HIV-1 on the gut microbiome are unclear. Initial cross-sectional studies provided contradictory associations between microbial richness and HIV serostatus and suggested shifts from *Bacteroides* to *Prevotella* predominance following HIV-1 infection, which have not been found in animal models or in studies matched for HIV-1 transmission groups. In two independent cohorts of HIV-1-infected subjects and HIV-1-negative controls in Barcelona (n = 156) and Stockholm (n = 84), men who have sex with men (MSM) predominantly belonged to the *Prevotella*-rich enterotype whereas most non-MSM subjects were enriched in *Bacteroides*, independently of HIV-1 status, and with only a limited contribution of diet effects. Moreover, MSM had a significantly richer and more diverse fecal microbiota than non-MSM individuals. After stratifying for sexual orientation, there was no solid evidence of an HIV-specific dysbiosis. However, HIV-1 infection remained consistently associated with reduced bacterial richness, the lowest bacterial richness being observed in subjects with a virological-immune discordant response to antiretroviral therapy. Our findings indicate that HIV gut microbiome studies must control for HIV risk factors and suggest interventions on gut bacterial richness as possible novel avenues to improve HIV-1-associated immune dysfunction.

## Highlights

• The fecal microbiota of gay men in Europe is systematically richer and has a distinct composition.
• HIV-1 infection is independently associated with reduced gut bacterial richness, more so in immune discordant subjects.
• Interventions to increase gut bacterial richness might offer novel avenues to improve HIV-1-associated immune dysfunction.

The human intestinal microbiota is essential for human health and well-being and is driven by genetic, lifestyle and environmental factors. Here, we show in two independent cohorts of HIV-1-infected subjects and HIV-1-negative controls in Europe that gay men often have a distinct composition of the human fecal microbiota, with increased microbial richness and diversity and enrichment in the *Prevotella* enterotype. This is independent of HIV-1 status, and with only a limited contribution of diet effects. After accounting for sexual orientation, however, HIV-1 infection remains associated to reduced bacterial richness, more so in subjects with suboptimal CD4 + T-cell count recovery under antiretroviral therapy. Future studies should evaluate if interventions to increase gut bacterial richness could improve HIV-associated immune dysfunction.

## 1 Introduction

The main clinical problems of people living with HIV (PLWH) in areas with adequate healthcare standards and continued antiretroviral therapy (ART) supply are increasingly related to premature aging (Paiardini and Müller-Trutwin, 2013). That is, a precocious development of type 2 diabetes, dislipidemia, cardiovascular diseases, osteoporosis and frailty syndrome. Such diseases have been related to structural or metabolic perturbations in the gut microbiota of non-HIV-infected subjects (Claesson et al., 2012, Koeth et al., 2013, Le Chatelier et al., 2013, Tang et al., 2013) whereas, in PLWH, have been linked to chronic inflammation, immune activation and endotoxemia (Brenchley et al., 2006, Douek, 2003, Sandler and Douek, 2012). Thus there is considerable interest in understanding the role of the human gut microbiome in HIV pathogenesis and, in particular, its ability to perpetuate chronic inflammation and foster immune senescence. This has immediate clinical implications because, in theory, it might be possible to gear the gut microbiota towards “healthier” equilibrium states with the host, which might allow, for example, to achieve faster immune reconstitution, improve vaccine responses or reduce HIV reservoirs.

However, although expectations are high, the HIV microbiome science is still at its early stages, and much remains to be known. Simple questions such as whether there is a consistent HIV-specific dysbiosis pattern, or which factors are relevant in shaping the microbiome in PLWH remain unanswered. Initial cross-sectional studies in humans have provided contradictory associations between microbial richness and HIV serostatus, and suggested shifts from *Bacteroides* to *Prevotella* predominance following HIV-1 infection (Lozupone et al., 2013, Vázquez-Castellano et al., 2015). Such shifts, however, have neither been found in animal models (Handley et al., 2012) nor in studies matching for HIV-1 risk groups (Yu et al., 2013). Conversely, large international studies in healthy populations have shown that at least in resource-rich countries, the gut microbiome forms a composition landscape with density peaks that can stratify the human population into enterotypes dominated by *Bacteroides*, *Prevotella* and *Ruminococcus*, respectively (Arumugam et al., 2011, Koren et al., 2013). The origin and clinical significance of such enterotypes is uncertain, but they have been linked to genetic (Goodrich et al., 2014), as well as to lifestyle (Clarke et al., 2014, David et al., 2013, Wu et al., 2011) and environmental factors (Modi et al., 2014, Sommer and Bäckhed, 2013), including long-term dietary patterns and exercise. Thereby, associations between *Prevotella* or *Bacteroides* and HIV infection might be easily confounded by other factors. Obtaining reliable information at this level is critical to advance our understanding of HIV pathogenesis, as well as to define the specific targets of novel therapeutic interventions on the human gut microbiome.

## 2 Methods

### 2.1 Study Design

This was a cross-sectional study in two independent European cohorts of HIV-1-infected subjects and HIV-negative controls. The study included one test cohort, one internal validation cohort and one external validation cohort (Supplementary Fig. 1).

The test cohort (BCN0) was enrolled in Barcelona, Catalonia, Spain, between January and December 2014. HIV-1 infected patients were recruited from HIV Clinics at the University Hospitals Germans Trias i Pujol and Vall d'Hebrón. HIV-1-negative controls were mainly recruited from an ongoing prospective cohort of HIV-negative MSM at risk of becoming infected by HIV-1 (Coll et al., 2015), who attend quarterly medical and counseling visits including HIV-1 testing (Alere Determine™ HIV-1/2 Ag/Ab Combo, Orlando, FL) at a community-based center for MSM in Barcelona (Meulbroek et al., 2013). Additional controls were HIV-1-negative partners from HIV-1-infected subjects attending the HIV clinics.

The inclusion criteria were: age within 18 and 60 years and body mass index (BMI) within 18.5 and 30. Exclusion criteria were: (a) any gross dietary deviation from a regular diet, or any specific regular diet, i.e., vegetarian, low-carb, etc.; (b) antibiotic use during the previous 3 months (with the exception of late presenters, who could receive antibiotics to treat opportunistic infections); (c) pregnancy or willingness to become pregnant; (d) current drug consumption or alcohol abuse; (e) any chronic digestive disease such as peptic ulcer, Crohn's disease, ulcerative colitis or coeliac disease; (f) any surgical resection of the intestines except for appendectomy; (g) any autoimmune disease; and (h) any symptomatic chronic liver disease or presence of hepatic insufficiency defined as a Child–Pugh C score. In addition, HIV-infected subjects were classified as elite controllers, viremic controllers, ART-naïve, early treated, late presenters, immune concordant or immune discordant (Supplementary methods).

The internal validation cohort (cohort BCN1) included individuals from BCN0 who provided a second fecal sample one month later.

Observations in Barcelona were externally validated in an independent observational cohort recruited at the HIV outpatient clinic, Karolinska University Hospital, Stockholm, Sweden (cohort STK). All HIV-1-infected patients in cohort STK were at least 18 years old, had been diagnosed with HIV-1 between one and 25 years earlier and were ART-naïve at the time of fecal sampling. Controls were healthy HIV-1-negative individuals matched by sex and age. Neither patients nor controls had been prescribed antibiotics or probiotics, or had had infectious diarrhea during the preceding two months.

### 2.2 Data Collection

Clinical and laboratory data from BCN0 and BCN1 were collected in a centralized database specifically designed for this study (OpenClinica™, © 2015 OpenClinica, LLC). The clinical evaluation was performed following a standardized questionnaire including: a checklist for fulfillment of inclusion and exclusion criteria, anthropometric data, age at study entry, age at HIV diagnosis, gender, ethnicity, city of residence, HIV risk group, history of allergies, antibiotic intake between 3 and 6 months before inclusion, frequency and consistency of feces, history of medical or surgical problems or interventions, present and previous ART, history of AIDS- and non-AIDS-related diseases, nadir and most recent CD4 + T-cell counts, HIV-1 RNA levels, history of sexually transmitted diseases and infection by the human papillomavirus (HPV), hepatitis B (HBV) or hepatitis C (HCV).

HIV-1 risk categories in our study were mutually excluding: male study participants who reported being MSM or referred insertive or receptive anal intercourse with other men were included in the MSM category, even if they also reported intravenous drug use or sex with women. Females and males not included in the MSM category reporting past intravenous drug use were classified as PWID. Heterosexual males or females not included in any of the previous 2 categories were classified as HTS. None of our study participants belonged to any other HIV-1 transmission category.

Study participants in Barcelona received a thorough dietary and nutritional assessment by a specialized dietitian/nutritionist using two standardized and validated questionnaires, i.e.: (a) a prospective dietary nutrient survey aimed at recording, as precisely as possible, any food, supplement or liquid intake during 3 to 5 consecutive days, including at least one weekend day, and (b) a recall of food portions taken per week, on average, during the last year.

Participants also went through a proctology evaluation by a specialized HIV physician/proctologist. In addition to visual inspection for anal or perianal lesions, HPV-related or not, the physician performed a rectal swab to rule out *Chlamydia trachomatis* and *Neisseria gonorrhoeae* infection using real-time PCR and an anal cytology. If the anal cytology reported an abnormal result, such as ASCUS (atypical squamous cells of undetermined significance), LSIL (low-grade squamous intraepithelial lesion) or HSIL (high-grade squamous intraepithelial lesion), the subject was properly treated and PCR typing of HPV was performed. No cases of anal cancer were detected.

In all study participants, we produced MiSeq™ 16S rRNA sequence data on fecal microbiomes and measured soluble plasma markers of enterocyte damage (intestinal fatty acid-binding protein, IFABP), microbial translocation [soluble CD14 (sCD14) and lipopolysaccharide binding protein (LBP)] and systemic inflammation [interleukin-6 (IL-6), C-reactive protein (CRP) and interferon-gamma-inducible protein-10 (IP-10)].

Study participants collected fecal samples in sterile fecal collection tubes the same day or the day before their clinical appointment, before the proctology exam, and following instructions pre-specified on standard operating procedures. If required, samples were stored at 4 °C overnight until DNA extraction. All samples collected in Barcelona were immediately extracted upon arrival to the laboratory. Additional aliquots were cryopreserved at − 80 °C for future studies. Samples collected in Stockholm were cryopreserved at − 80 °C and shipped on dry ice in batch to the IrsiCaixa AIDS Research Institute, where they were extracted, amplified, sequenced and analyzed using the exact same procedures applied to the Barcelona samples. The lag times to freezing were always < 36 h and no particular chemical stabilizers were added to samples used for the analyses presented here. Fecal sample collection procedures were the same for cases and controls.

Detailed descriptions of the wet-lab procedures and the ecological and statistical analyses of the microbiome, soluble plasma markers and the nutritional assessment are available in the Supplementary methods section.

### 2.3 Ethics & Community Involvement

The study was reviewed and approved by the Institutional Review Boards of the Hospital Universitari Germans Trias i Pujol (reference PI-13-046) and the Hospital Vall d'Hebrón (reference PR(AG)109/2014). The Stockholm study cohort was approved by the Regional Ethical Committee (Stockholm, Sweden, Dnr 2009-1485-31-3). All participants provided written informed consent in accordance with the World Medical Association Declaration of Helsinki. The study concept, design, patient information and results were discussed with the IrsiCaixa's Community Advisory Committee, who also provided input on the presentation and dissemination of study results (Supplementary methods).

### 2.4 Sequence and Data Availability

Raw Illumina MiSeq sequences and study metadata were deposited in the National Center for Biotechnology Information — NCBI repository (Bioproject accession number: PRJNA307231, SRA accession number: SRP068240).

### 2.5 Financial Support and Role of the Funding Sources

This study was mainly funded through philanthropy and private donations, which had no influence on its contents. Funds were obtained from a personal donation from Mr. Rafael Punter, the *Gala contra la SIDA* 2013 and 2014 editions, and the *Nit per la Recerca a la Catalunya Central* 2015 edition. M.R. is funded through a FI-DGR grant (FI-B00184) from *Agència de Gestió d*'*Ajuts Universitaris i de Recerca* (AGAUR) at the *Secretaria d*'*Universitats i Recerca del Departament d*'*Economia i Coneixement de la Generalitat de Catalunya*. Y.G. is supported through a post-doctoral grant from the *Fundación Paideia Galiza*. M.C. is funded through the *Red de Investigación en SIDA*, RD12/0017/0002 as part of the Plan Nacional R + D + I and cofinanced by the *Instituto de Salud Carlos III* (ISCIII)-*Subdirección General de Evaluación y el Fondo Europeo de Desarrollo Regional* (FEDER). J.R. is supported through a grant for doctoral studies from Noel Alimentaria to the University of Vic (UVic-UCC). B.M. is a Joan Rodés investigator from the ISCIII (JR13/00024), Madrid, Spain. M.L.C. is funded through the grant MTM2012-38067-C02-02, Spanish Ministry of Economy and Competitiveness, Spain. PB, FH and GZ are supported by the European Molecular Biology Laboratory. F.H. was funded from the European Union's Horizon 2020 research and innovation program under the Marie Skłodowska-Curie grant agreement no. 600375. P.B. acknowledges the European Research Council grant Cancerbiome, reference 268985. The sponsors of the study had no role in study design, data collection, data analysis, data interpretation, or writing of the report. The corresponding author had full access to all study data, and had final responsibility for the decision to submit for publication.

## 3 Results

### 3.1 Study Subjects

The study included 240 individuals, 156 in Barcelona (Table 1) and 84 in Stockholm (Table 2). The test cohort BCN0 comprised 129 (82.7%) HIV-1-infected and 27 (17.3%) HIV-negative subjects. The internal validation cohort BCN1 included 110 individuals, 87 HIV-1-infected (79.1%) and 23 non-HIV-infected (20.9%). The external validation cohort STK had 77 HIV-1-infected (91.6%) and 7 non-HIV-infected individuals (8.4%). In Barcelona, the median age of study participants was 43 years and their median body mass index was 23.8 kg/m^2^. Eighty percent of subjects were men, mostly from Caucasian ethnicity. Sixty-four percent of all subjects were MSM, 26% HTS and 10% PWID. There were 8 (5.1%) elite controllers, 11 (7.1%) viremic controllers, 15 (9.6%) ART-naïve, 13 (8.3%) early-treated, 53 (34.1%) immune concordant, 18 (11.5%) immune discordant, and 11 (7.1%) late presenters. HIV-1-infected subjects were slightly older and were more likely to be HBV and HCV positive than HIV-negative controls. Groups were well balanced in all other factors. In Stockholm, 60% of subjects were men; 23% were MSM, 66% HTS and 11% PWID. Only half were nationals from Scandinavian countries; 62% individuals were Caucasian and 33% were Black.

### 3.2 Richness and Diversity of the Fecal Microbiota

**Fig. 1:**
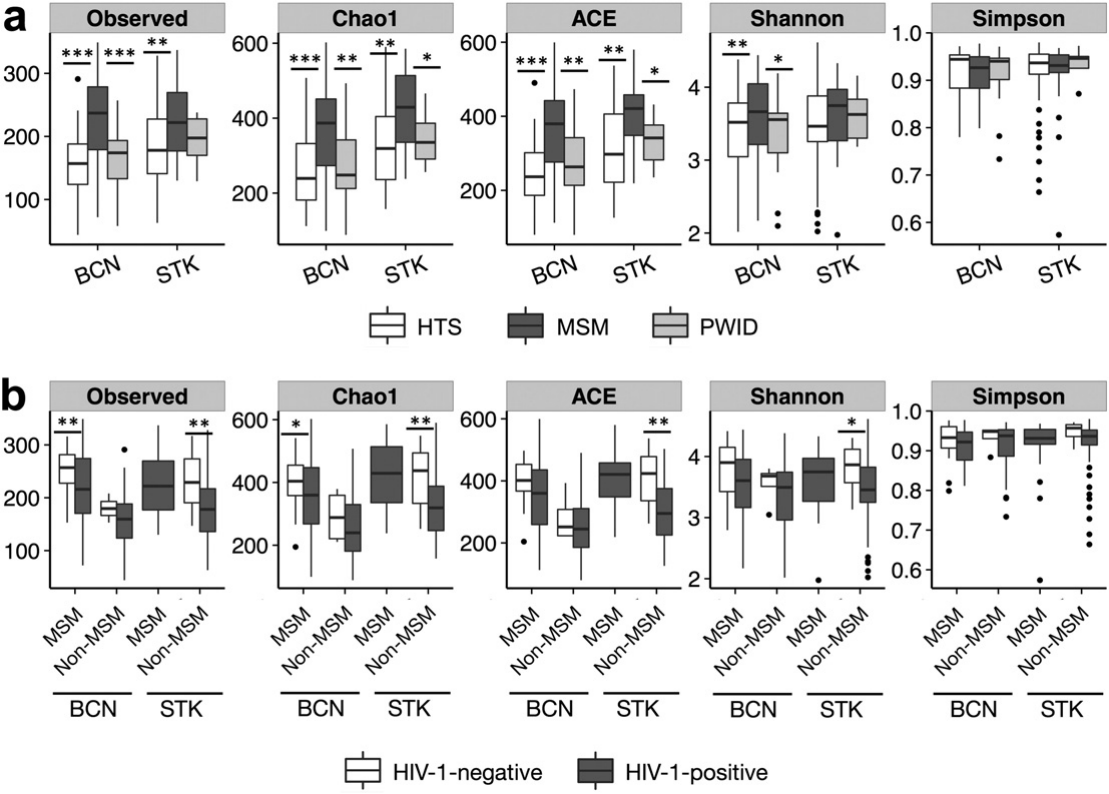
Both HIV transmission group and HIV-1 infection are linked to the human fecal microbiome richness and diversity. a) The highest richness and diversity in human fecal microbiota were observed in men who have sex with men (MSM). There were no differences between heterosexual subjects (HTS) and people who acquired HIV-1 infection through intravenous drug use (PWID). Kruskal–Wallis p-values in a were adjusted for multiple comparisons using the Benjamini–Hochberg method. b) HIV-1 infection was associated with significant reductions in fecal human microbiome richness after stratifying for sexual preference. Comparisons were made with the Wilcoxon rank sum test with continuity correction. All alpha diversity findings were consistent in Barcelona (test cohort, month 0) (BCN0) and Stockhom (STK). Identical results were found in BCN at month 1 (Supplementary Fig. 2) and when using an independent sequence analysis pipeline (Supplementary Fig. 14). Note: “Simpson” refers to 1-Simpson index. The remaining ecological index names are self-explanatory. *p < 0.1, **p < 0.05, ***p < 0.001.

**Fig. 2:**
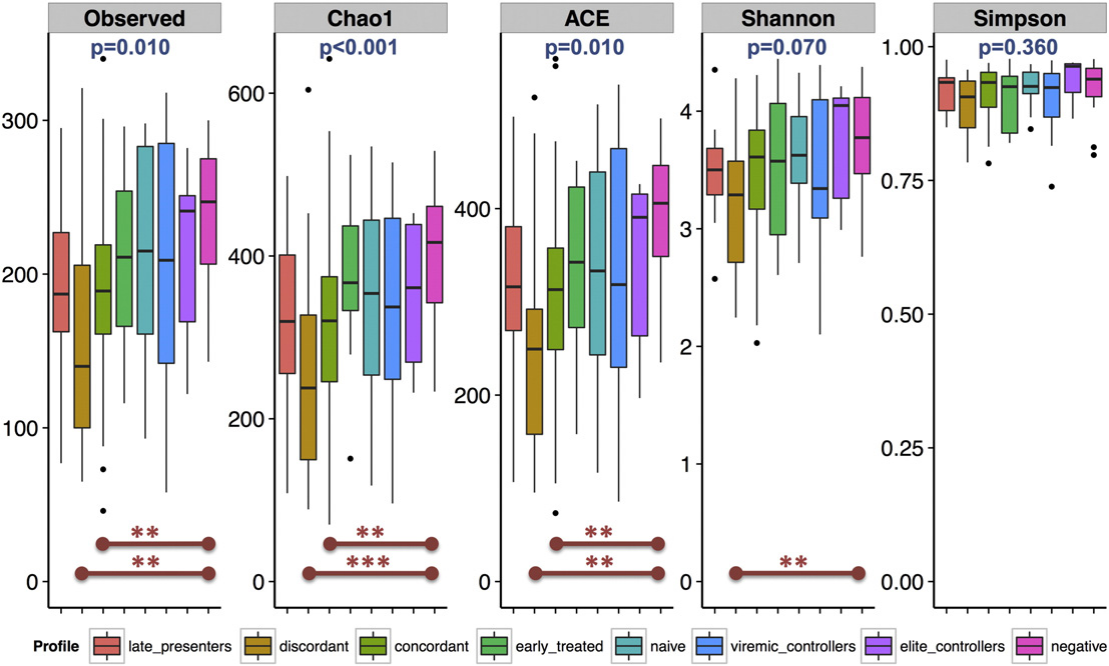
Alpha diversity by HIV-1 phenotype. HIV-1-infected subjects with an immune discordant phenotype (i.e. those who do not recover CD4 + counts > 300 cells/mm^3^ despite at least 2 years of effective antiretroviral therapy) had the lowest microbiome richness of all HIV-1 phenotypes. Individuals with an immune concordant phenotype (i.e., those achieving CD4 + count reconstitution > 500 cells/mm^3^ on antiretroviral therapy) also had lower microbiome richness than HIV-1-negative individuals, but not as low as immune discordant subjects. “Simpson” refers to 1-Simpson index. The remaining ecological index names are self-explanatory. Comparisons were done using a Kruskal–Wallis test including post-hoc pairwise analyses. Benjamini–Hochberg-adjusted p-values are shown at the top of each index; for post-hoc pairwise comparisons: *p < 0.1, **p < 0.05, ***p < 0.001.

The fecal microbiota was significantly richer and more diverse in MSM than non-MSM individuals in both cities, also after correcting for multiple comparisons (Fig. 1, Supplementary Figs. 2 and 14). This indicated that the measurement of the effect of HIV-1 on gut microbial richness and diversity had to take HIV transmission group into account. After stratifying for MSM vs. non-MSM, HIV-1 infection remained consistently associated with reduced bacterial richness (15% to 30% reduction relative to HIV-negative individuals) in both groups and both cities (Fig. 1, Supplementary Figs. 2 and 14). In the Barcelona cohort, the lowest microbial richness and diversity was observed among HIV-1-infected individuals with an immune-virological discordant phenotype (Fig. 2). Subjects with an immune-virological concordant phenotype had higher microbial richness than immune discordant individuals, but, nevertheless, still showed reduced microbial richness relative to HIV-negative controls, suggesting that despite adequate immune recovery [median (IQR) CD4 + T-cell counts: 761 (640, 932) cells/mm^3^] at the time of testing, ART had not been able to fully normalize microbial richness.

### 3.3 Bacterial Composition of the Fecal Microbiota

**Fig. 3:**
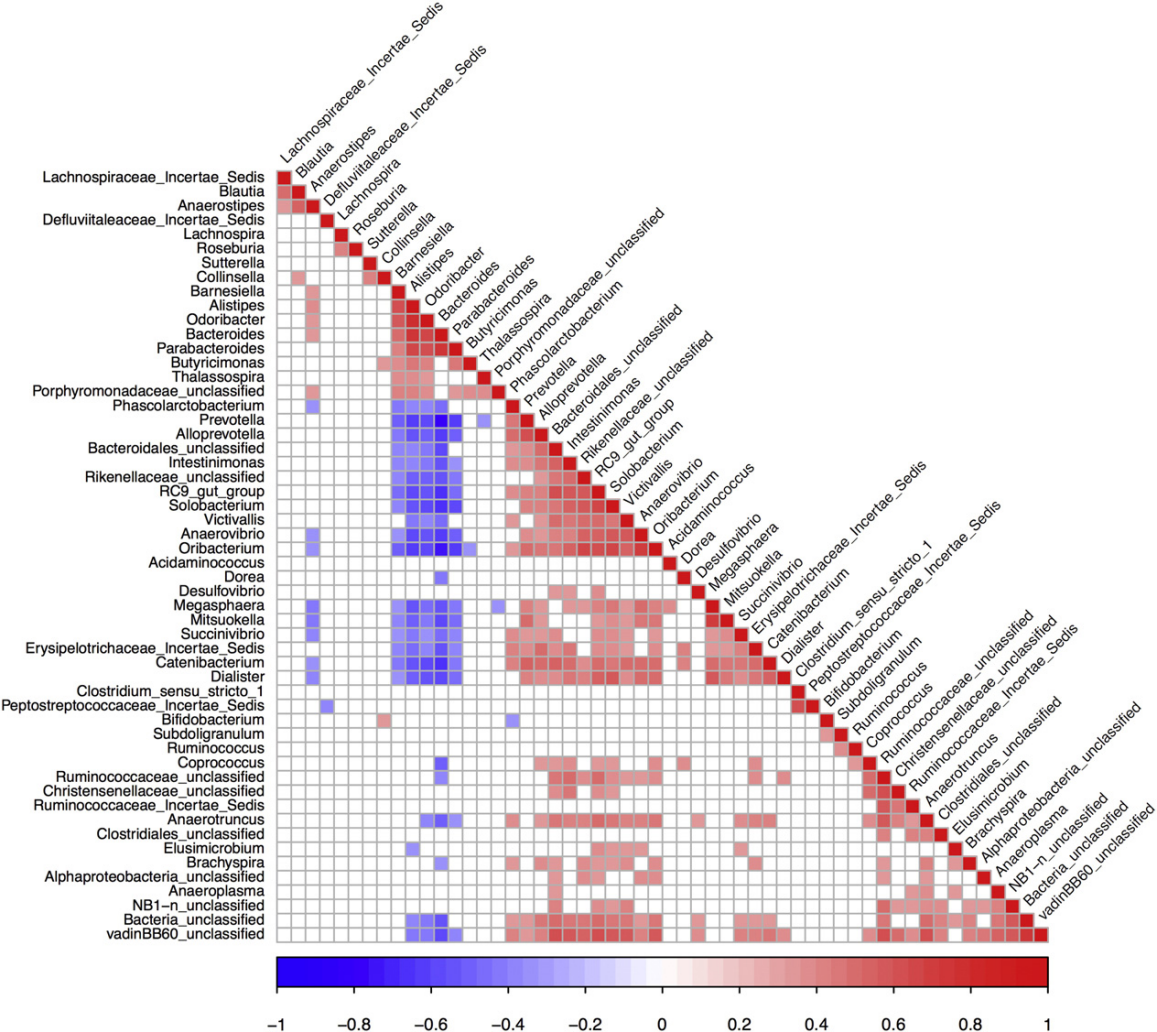
Spearman correlation by genus abundance. Only significant values (Holm's-corrected p < 0.05) are shown. The plot confirms previous observations, i.e.: a) strong positive correlations between *Bacteroides*, *Parabacteroides*, *Barnesiella*, *Alistipes* and *Odoribacter*, b) strong positive correlations between *Prevotella*, *Alloprevotella*, *Mitsuokella* and *Intestinimonas*, among others, and c, strong inverse correlations between the groups including *Prevotella* and *Bacteroides*.

**Fig. 4:**
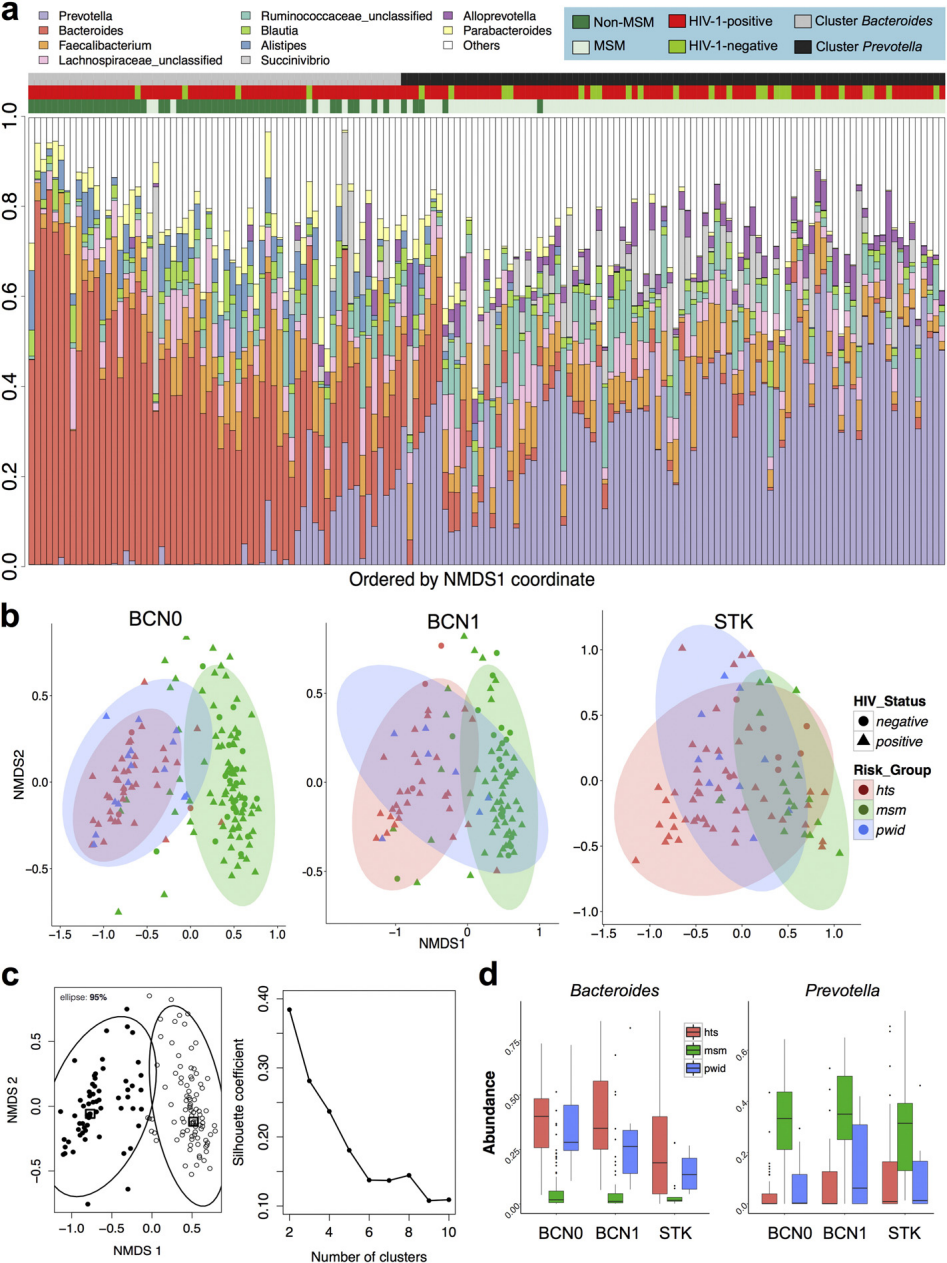
The bacterial genus composition of the human fecal microbiome is mainly linked to HIV transmission group. a) The bacterial genus composition of the fecal microbiota in the Barcelona test dataset (BCN0) was largely determined by HIV transmission group, with MSM being enriched in the *Prevotella* cluster and non-MSM in the *Bacteroides* cluster. Genera with mean abundance of at least 2% across all samples are represented in colors; those with < 2% abundance are grouped into the category “Others”. Each column represents one individual. A similar plot for the Stockholm cohort is shown in Supplementary Fig. 12. b) Non-metric multidimensional scaling (NMDS) ordination plots of Bray–Curtis distances showing that microbiomes in the BCN0, BCN1 and STK datasets mainly cluster by HIV transmission group (MSM vs. non-MSM) rather than by HIV serostatus. Ellipses include 95% of samples. Similar plots using other distances are shown in Supplementary Figs. 8 to 10. c) Partitioning around medoids (PAM) analysis of the BCN0 dataset showing this population structure in this dataset is better explained by 2 rather than more clusters, with reasonable Silhouette support. This information was used to define the *Bacteroides* and *Prevotella* clusters in our study. d) Abundance box plots showing that MSM were enriched in *Prevotella* and non-MSM (HTS or PWID) were enriched in *Bacteroides*. Comparisons between MSM and the non-MSM categories were always highly significant (p < 0.001) after adjusting for multiple comparisons using the Benjamini–Hochberg method. Plots of all genera showing significant differences between MSM and non-MSM categories are shown in the Supplementary Figs. 13 to 15.

Clustering of the fecal microbiomes in BCN0 and STK using a partitioning around medoids (PAM) algorithm suggested the presence of at least 2 clusters of fecal microbiomes in both cities (Fig. 4c). Such clusters were enriched either in *Bacteroides* or *Prevotella*, and had a similar bacterial composition to the corresponding previously described enterotypes (Arumugam et al., 2011, Koren et al., 2013) (Supplementary Fig. 3). As expected from previous work on gut enterotypes, there were strong positive correlations between the genus *Bacteroides* and *Parabacteroides*, *Barnesiella*, *Alistipes* and *Odoribacter*, as well as between *Prevotella* and *Alloprevotella*, *Catenibacterium*, *Mitsuokella* and *Intestinimonas*, among others (Fig. 3), highlighting that differences between the groups extended beyond a single genus. The genera correlating with *Prevotella* were negatively correlated with *Bacteroides* and vice versa. Moreover, the microbiomes of the *Bacteroides* and *Prevotella* clusters showed remarkably different functional profiles (Supplementary Figs. 4 and 5), also in agreement with previous enterotype descriptions (Arumugam et al., 2011).

### 3.4 Factors Associated With the Fecal Microbiota Composition

We explored variables potentially influencing the composition of the fecal microbiomes, according to a univariate ADONIS test of ecological distance and found possible effects of HIV-1 risk group, gender, feces consistency, place of residency, ethnicity, HIV-1 serostatus and altered abdominal transit (Supplementary Table 1). However, only the HIV-1 risk group retained statistical significance in a multivariate ADONIS analysis with terms added sequentially (R^2^: 0.373, p < 0.001).

Fecal microbiomes in BCN0, BCN1 and STK clustered by HIV transmission group rather than by HIV-1 serostatus, using either Bray–Curtis (Fig. 4b) or other ecological distances (Supplementary Figs. 6 to 8). Although a few individuals showed marked differences between the two time points, fecal microbiota ordination was highly concordant between BCN0 and BCN1 (Procrustes m2 = 0.3475, PROTEST p = 0.001) (Supplementary Fig. 9), indicating that differences in microbial ordination were not due to random variation. The fecal microbiota composition in both BCN0 and STK significantly differed by HIV transmission group, with MSM and non-MSM subjects mostly belonging to the *Prevotella* and *Bacteroides* clusters, respectively (Fig. 4a and 4d and Supplementary Figs. 10 to 14). Alpha and beta diversity and genus abundance analyses were reproducible using a different analysis pipeline (Hildebrand et al., 2014) (Supplementary Fig. 14 and Supplementary methods).

In an analysis accounting for the potential interdependency of sexual preference and HIV-1 serostatus (LEfSe) (Segata et al., 2011), there were consistent differences in both cities only by sexual preference group, with enrichment of *Prevotella*, *Alloprevotella*, *Succinvibrio*, *Dorea*, RC 9 gut group, *Desulfovibrio*, *Phascolarctobacterium* and unclassified *Bacteroidales* in MSM, and enrichment in *Bacteroides*, *Odoribacter* and *Barnesiella* in non-MSM individuals (Supplementary Fig. 15).

### 3.5 Strength of the Associations

**Fig. 5:**
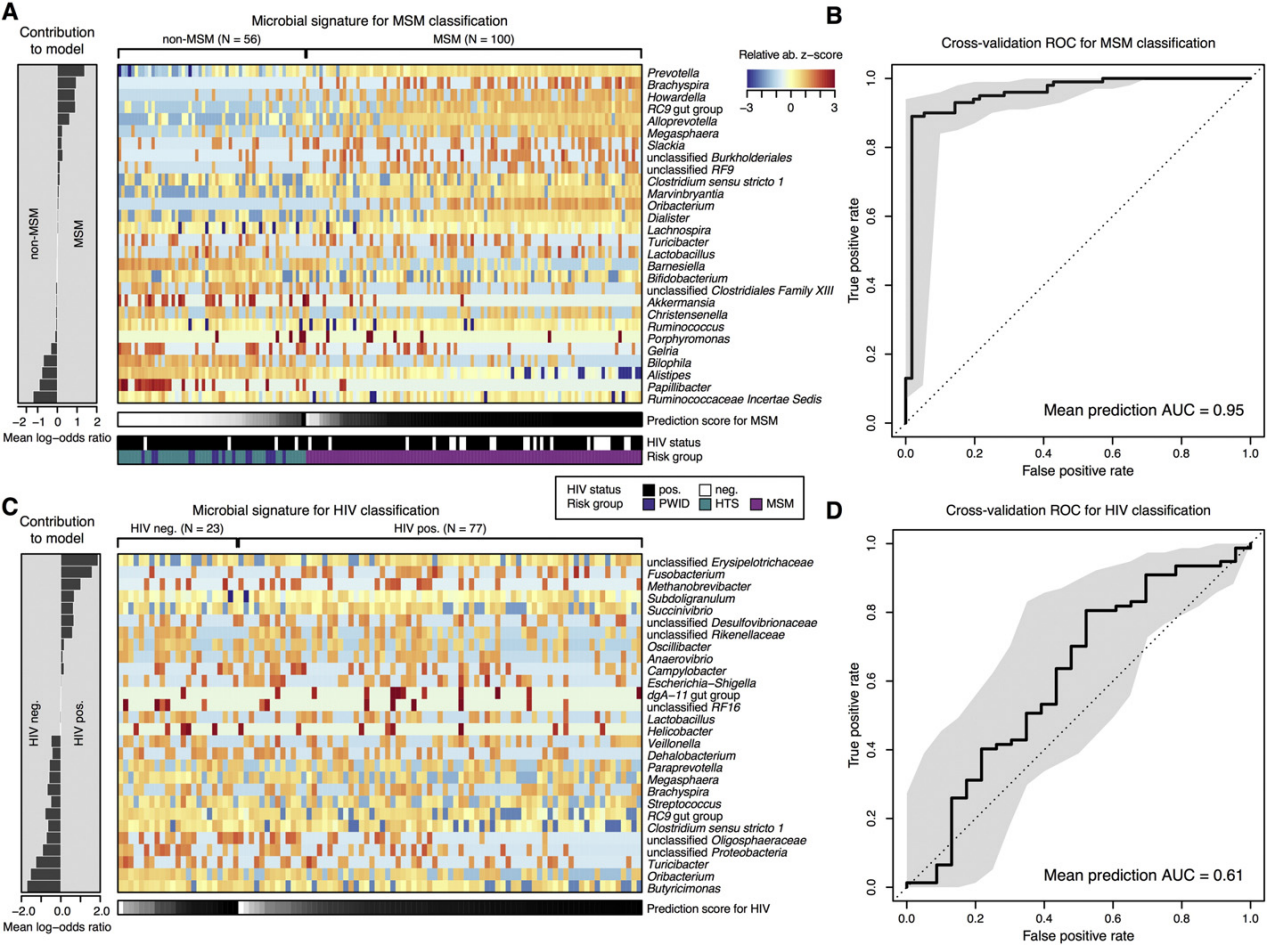
Global microbiota classifier by sexual preference group and HIV-1 status. A and C) Relative abundances of 28 gut microbial genera collectively associated with MSM and HIV-1 infection, respectively, are displayed as heatmap of log-abundance z-scores with the direction of association indicated to the left. To avoid confounding by sexual preference, the HIV-1 classifier only includes MSM subjects. The mean contribution of each marker species to the classification is shown to the left (bars correspond to log-odds ratio in logistic regression). Below each heatmap the classification score of the microbial signature from cross-validation is shown as gray scale. HIV-1 status and HIV-1 risk group are color-coded below the first heatmap (see color key). B and D) Cross-validation accuracy of the microbiota classifier is depicted as receiver–operator-characteristic (ROC) curve summarizing mean test predictions made in ten times resampled tenfold cross-validation with the area under the curve (AUC) indicated inside each plot. As shown, there was a strong association between the global microbiome genus composition and sexual orientation, whereas the association with HIV-1 infection was much weaker and of uncertain significance.

To quantify the strength of the association between HIV transmission group, HIV serostatus and global fecal microbiota composition, we applied a previously validated global microbiota classification concept based on LASSO regression (Zeller et al., 2014) to our BCN0 dataset. Cross-validation accuracy was extraordinarily high for sexual preference group (mean AUC = 95%), confirming a different fecal microbiota composition in MSM and non-MSM individuals (Fig. 5). In contrast, HIV-1 status was not associated with consistent changes in the global fecal microbiota composition at the genus level, suggesting that the reduction in microbial richness observed in HIV-infected individuals was not genus-specific.

**Fig. 6:**
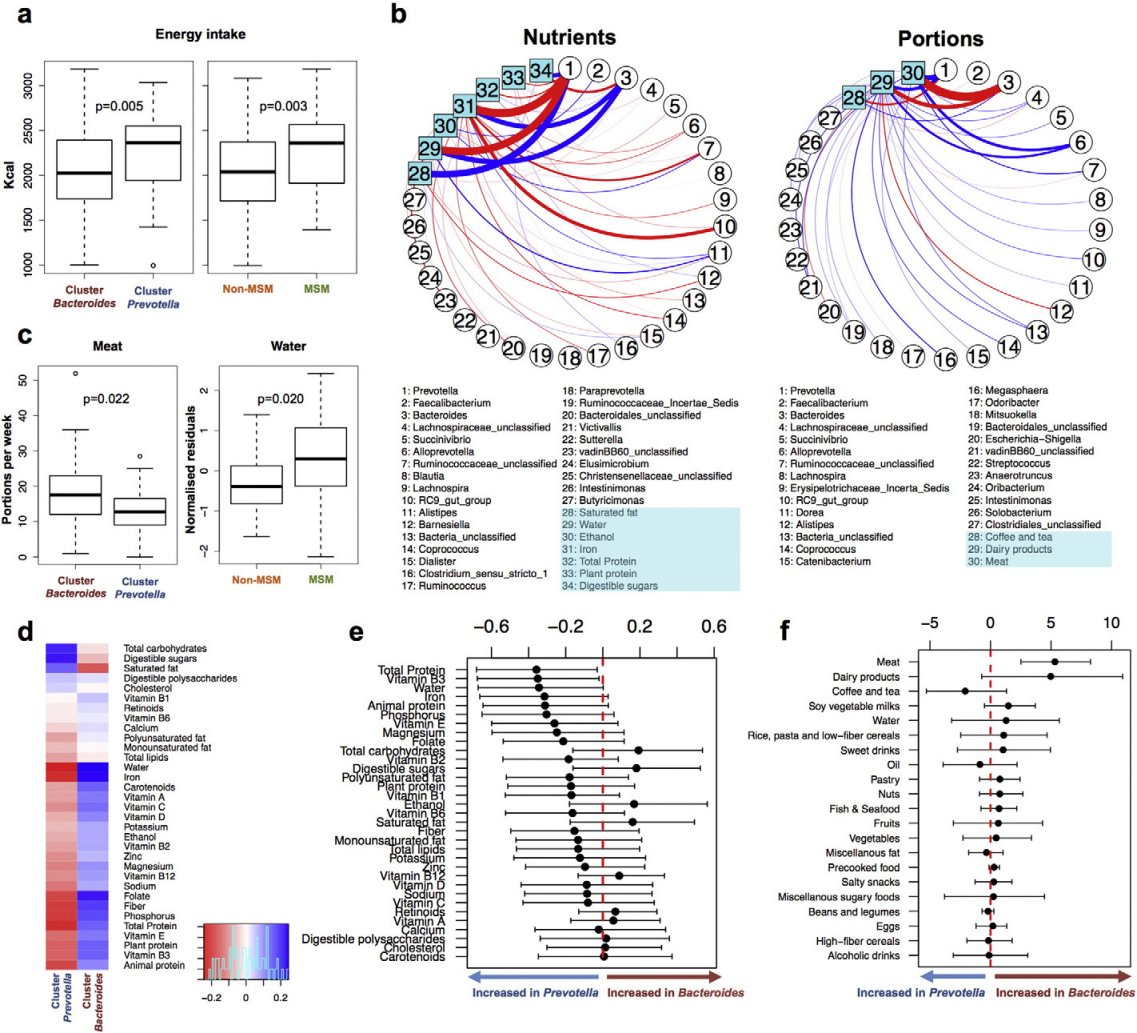
Limited effect of diet on the composition of the microbiome. a) Subjects belonging to the *Prevotella* cluster and men who had sex with men (MSM) had significantly higher total energy intake. Therefore, all subsequent nutritional analyses were normalized for this factor. b) Main associations between bacterial genera, normalized amounts of nutrients (left) and food portions (right), according to a Dirichlet multinomial regression model. Positive and negative associations are shown in red and blue, respectively. Line thickness is proportional to the strength of the association. c) Of all links identified by the Dirichlet approach, the only significant differences between groups after adjusting for multiple comparisons (Benjamini–Hochberg FDR < 0.1) were increased consumption of meat in the cluster *Bacteroides* and increased intake of dietary water in MSM. d) Spearman correlations between normalized amounts of nutrients and Bray–Curtis distance to the furthest subject in the opposite cluster. Negative correlations imply increased amounts of nutrient with shorter distance to each cluster. Therefore, values in red and blue represent increased and decreased amounts of nutrients within each cluster, respectively. Although, in general, the direction of the correlations was concordant with previous publications, note the small effect sizes (R^2^ below the color key). None of the comparisons were statistically significant after correction for multiple comparisons (Benjamini–Hochberg FDR < 0.1); Permanova p = 0.20 for overall differences between clusters. e, f) Mean and 95% confidence intervals for the differences between clusters in consumption of nutrients (e) and portions of food (f). Comparisons were significant if the 95 confidence interval did not cross 0 (dashed red line).

Relative to non-MSM subjects, MSM were younger, were more likely to live in Barcelona City, reported softer fecal consistency, and were less likely to be infected with HBV and HCV (Supplementary Table 2). However, none of these factors among others were likely to confound the previous LASSO models (Supplementary Fig. 16). Although long-term dietary patterns have been linked to alternative enterotype states (Wu et al., 2011), the effect of diet on microbiota composition was limited in our setting (Fig. 6 and Supplementary Fig. 17) and none of the diet components was selected by multivariate LASSO regression as a consistent predictor of microbiota clustering.

### 3.6 Consequences on Enterocyte Damage, Microbial Translocation and Systemic Inflammation

Markers of enterocyte damage, microbial translocation and systemic inflammation followed an overall predictable response across different HIV phenotypes (Brenchley and Douek, 2012), being generally higher in immune discordant and late presenters (Supplementary Figs. 18 and 19). However, they did not differ between the *Bacteroides* or *Prevotella* clusters or between MSM and non-MSM individuals.

## 4 Discussion

In two independent European cohorts with different ethnic and cultural background, the fecal microbiota of MSM was consistently richer and more diverse than that of non-MSM subjects, and was systematically enriched in genera from the *Prevotella* enterotype. The strength of such association was unusually high, reaching 95% accuracy in a microbial composition-based classifier. These findings have important implications for HIV microbiome science. To our knowledge, this is the first evidence that, in addition to genetic (Goodrich et al., 2014), lifestyle (Clarke et al., 2014, David et al., 2013, Wu et al., 2011) and environmental factors (Modi et al., 2014, Sommer and Bäckhed, 2013), factors related with sexual preference might also affect the gut microbiota composition.

Based on our findings, previous associations between HIV infection and *Prevotella* might be explained by enrichment of HIV-infected groups by MSM relative to HIV-negative controls selected from hospital or research staff, gut biopsy donors, or college students (Lozupone et al., 2013, Mutlu et al., 2014, Vázquez-Castellano et al., 2015). Contradictory associations between HIV infection and microbial richness could also be affected by unbalances in the proportion of MSM between groups. Of note, a selection bias as such could also affect the interpretation of in silico inferences on bacterial metabolism, or even direct metabolomic or metatranscriptomic measurements, which also rely on bacterial composition.

In concordance with data from animal models (Handley et al., 2012) and studies matching for HIV risk factors (Yu et al., 2013), we were unable to identify a consistent HIV-specific fecal dysbiosis pattern after stratifying for HIV transmission group. Yet, HIV-1 infection remained associated with reduced bacterial richness independently of sexual orientation, indicating that the most evident hallmark of HIV infection on the gut microbiome is, like in other intestinal inflammatory diseases (Manichanh et al., 2012), a reduction in bacterial richness. In line with previous observations linking bacterial richness with immune dysfunction (Nowak et al., 2015), the lowest bacterial richness was found in immune discordant subjects, followed by immune concordant individuals with adequate immune recovery on ART. Conversely, bacterial richness was conserved in subjects initiating ART during the first 6 months of HIV infection, as well as in ART-naïve individuals with > 500 CD4 + counts/mm^3^, suggesting that early ART initiation might help to preserve gut microbial richness.

The strong epidemiological association of fecal microbiota composition with sexual orientation in two independent cities is yet to be translated into specific mechanisms. We ruled out multiple confounders and only found a limited effect of diet in our setting. We did not collect information on exercise, but exercise has been linked to fecal microbiota composition in athletes (Clarke et al., 2014) and even in them diet plays an important role. A formal assessment of the socioeconomic status of our patients was out of the scope of this work, although based on our findings, rigorous studies assessing the role of socioeconomic status in the fecal microbiota composition are needed. Non-MSM subjects in our study were older and more likely to be co-infected with HBV and HCV than MSM, reflecting current trends of the HIV epidemic in Europe, i.e.: most new HIV-1 infections occur in young MSM who rarely use intravenous drugs. Fecal consistency was also softer in MSM than in non-MSM subjects, which, indirectly, might reflect better overall health habits, including a healthier diet, higher water consumption and physical activity. However, none of these factors, nor ethnicity, achieved a significant weight in LASSO models.

Further studies are needed to evaluate the existence of ecological adaptations of commensal bacteria to changes in gut mucosa induced by sexual practices. Populations of commensal bacteria are controlled by substrate competition and glycan availability (Koropatkin et al., 2012) and several factors might affect distal colorectal mucosa, including hyperosmolar substances like semen or certain lubricants (Fuchs et al., 2007, McGowan, 2012), colorectal cleansing or use of sexual toys. Longitudinal studies should also clarify if the observed association is stable over time, and if it varies according to the number of sexual partners (i.e., long-term single relationships versus frequent partner exchange) or by insertive versus receptive anal sex. It is also important to clarify if the observed association remains in heterosexual women who engage in receptive anal sex and if increased microbiota richness can be related to person-to-person transmission of commensal bacteria. Future studies should also investigate if the observed association has implications for transmission of infectious agents, including HIV-1. We did not find an association between fecal microbiome and HBV, HCV, syphillis or rectal HPV, *C. trachomatis* or *N. gonorrhoeae* infections, but did not evaluate HSV-2 infection. In our study, the observed association between sexual orientation and microbiota composition did not translate into gross differences in terms of systemic inflammation or microbial translocation. Shotgun metagenomic analyses of bacterial species and richness, as well as the virome and perhaps the mycobiome, in clinical trials balanced by HIV risk factors might provide novel clues as to the impact of HIV infection on the gut microbiome.

In conclusion, the fecal microbiota of gay men in Europe is richer and has a distinct composition. However, HIV-1 infection remains independently associated with reduced bacterial richness. This offers new avenues for therapeutic interventions on the gut microbiome which might improve HIV-associated immune dysfunction.

## Author Contributions

R.P., M.N., P.N., A.S., J.B. and B.C. conceived and designed the study. R.P., I.B., B.M., E.N., J.Co., J.S., A.T., J.N., C.B. and B.C in Barcelona and P.N., and A.S., in Stockholm, recruited the study participants and performed their clinical evaluations. C.E. performed the dietary assessment. G.S. and J.C. performed the proctology studies. C.H. coordinated the study logistics including the fulfillment of all ethical and legal requirements of the study as member of the Contract Research Organization overseeing the study, in coordination with R.P. Fecal 16S rDNA was extracted, amplified and sequenced by M.P, M.C., M.R. and R.B. under the supervision of M.N. and R.P. M.R., Y.G, J.R., C.R., F.H. and G.Z. performed the bioinformatic and statistical analyses of the 16S rDNA data, with the supervision of M.N., M.L.C., P.B., F.G. and R.P. M.C. performed the inflammation analyses under the supervision of J.Ca., J.B and R.P. J.R., did the statistical analyses of the relationship between the microbiota and inflammation and diet, under the supervision of M.N., M.L.C., F.G. and R.P. F.H and G.Z. performed the multivariate analysis of factors determining microbiota clusters and ran the confirmatory analyses with the independent sequence analysis pipeline LotuS, under supervision of P.B. R.P. wrote the paper, which was reviewed, edited and approved by all authors.

## Conflicts of Interest

The authors declare that they have no conflicts of interest.

## Figures and Tables

**Table 1:** Baseline chacteristics of subjects in the Barcelona test dataset (BCN0).

		Full dataset	HIV-1 positive	HIV-1 negative	p-Value	
No. of subjects		156	129	27
Age (years)^a^		43 (35, 51)	44 (36, 52)	37 (34, 44)	0.021	
Gender	Male	124 (79.5%)	101 (78.3%)	23 (85.2%)	0.076	0.600
Female	31 (19.9%)	28 (21.7%)	3 (11.1%)	0.291
Transgender	1 (0.6%)	0	1 (3.7%)	0.173
Ethnicity	Asiatic	1 (0.6%)	1 (0.8%)	0	0.900	1
Caucasian	124 (79.5%)	101 (78.3%)	23 (85.2%)	0.600
Hispanic–Latino	28 (18%)	24 (18.6%)	4 (14.8%)	0.786
Others	3 (1.9%)	3 (2.3%)	0	1
Risk group	HTS	41 (26.3%)	37 (28.7%)	4 (14.8%)	0.027	0.156
MSM	100 (64.1%)	77 (59.7%)	23 (85.2%)	0.014
PWID	15 (9.6%)	15 (11.6%)	0	0.075
Residency	Barcelona	51 (32.7%)	36 (27.9%)	15 (55.6%)	0.058	0.007
BCN Met	56 (35.8%)	50 (38.8%)	6 (22.2%)	0.125
Outside BCN Met	38 (24.4%)	33 (25.6%)	5 (18.5%)	0.622
na	11 (7.1%)	10 (7.7%)	1 (3.7%)	0.691
Profile	Late presenter	11 (7.1%)	11 (8.5%)	0	–	–
Discordant	18 (11.5%)	18 (14%)	0	–
Concordant	53 (34%)	53 (41.1%)	0	–
Early-treated	13 (8.3%)	13 (10.1%)	0	–
Naïve	15 (9.6%)	15 (11.6%)	0	–
Viremic control	11 (7.1%)	11 (8.5%)	0	–
Elite control	8 (5.1%)	8 (6.2%)	0	–
HIV-1 negative	27 (17.3%)	0	27 (100%)	–
BMI (kg/m^2^)^a^		23.8 (22, 26)	23.8 (22, 26)	24.9 (22, 27)	0.469	
Allergy	No	122 (78.2%)	101 (78.3%)	21 (77.8%)	0.205	1
Yes	30 (19.2%)	26 (20.2%)	4 (14.8%)	0.603
na	4 (2.6%)	2 (1.5%)	2 (7.4%)	0.138
ATB during the previous 3–6 months		35 (22.4%)	32 (24%)	4 (14.8%)	0.446	
Fecal consistency	Hard	56 (35.9%)	44 (34.1%)	12 (44.4%)	0.535	0.378
Soft	91 (58.3%)	77 (59.7%)	14 (51.9%)	0.521
Liquid	5 (3.2%)	5 (3.9%)	0	0.588
na	4 (2.6%)	3 (2.3%)	1 (3.7%)	0.536
Abdominal transit alterations	Yes	23 (14.7%)	22 (17.1%)	1 (3.7%)	0.089	0.134
No	127 (81.4%)	103 (79.8%)	24 (88.9%)	0.414
na	6 (3.9%)	4 (3.1%)	2 (7.4%)	0.277
Defecation frequency (per day)	1	88 (56.4%)	70 (54.3%)	18 (66.7%)	0.669	0.288
2	47 (30.1%)	40 (31%)	7 (25.9%)	0.653
3	12 (7.7%)	11 (8.5%)	1 (3.7%)	0.692
4	5 (3.2%)	5 (3.9%)	0	0.588
na	4 (2.6%)	3 (2.3%)	1 (3.7%)	0.536
HBV co-infection	Positive	19 (12.2%)	19 (14.7%)	0	0.054	0.045
Negative	112 (71.8%)	91 (70.6%)	21 (77.8%)	0.638
na	25 (16%)	19 (14.7%)	6 (22.2%)	0.386
HCV co-infection	Positive	24 (15.4%)	24 (18.6%)	0	0.013	0.015
Negative	120 (76.9%)	94 (72.9%)	26 (96.3%)	0.005
na	12 (7.7%)	11 (8.5%)	1 (3.7%)	0.692
Syphilis serology	Positive	21 (13.5%)	20 (15.5%)	1 (3.7%)	0.262	0.128
Negative	116 (74.3%)	93 (72.1%)	23 (85.2%)	0.225
na	19 (12.2%)	16 (12.4%)	3 (11.1%)	1
PCR *Chlamydia trachomatis*	Positive	9 (5.8%)	9 (7.0%)	0	0.161	0.360
Negative	115 (73.7%)	91 (70.5%)	24 (88.9%)	0.055
na	32 (20.5%)	29 (22.5%)	3 (11.1%)	0.293
PCR *Neisseria gonorrhoeae*	Positive	0	0	0	0.109	
Negative	125 (80.1%)	100 (77.5%)	25 (92.6%)
na	31 (19.9%)	29 (22.5%)	2 (7.4%)
PCR human papilloma virus	Yes	72 (46.2%)	61 (47.3%)	11 (40.7%)	0.613	0.671
No	83 (53.2%)	67 (51.9%)	16 (59.3%)	0.530
na	1 (0.6%)	1 (0.8%)	0	1
Anal cytology	ASCUS	22 (14.1%)	17 (13.2%)	5 (18.5%)	0.664	0.542
HSIL	7 (4.5%)	7 (5.4%)	0	0.605
LSIL	30 (19.2%)	26 (20.2%)	4 (14.8%)	0.603
Normal	80 (51.3%)	64 (49.6%)	16 (59.3%)	0.402
na	17 (10.9%)	15 (11.6%)	2 (7.4%)	0.738
CD4 + T-cell count (cells/mm^3^)^a^	All	–	700 (462, 860)	–	–	–
Late presenters	–	100 (33, 189)	–	–
Discordant	–	263 (223, 287)	–	–
Concordant	–	761 (640, 932)	–	–
Early-treated	–	785 (506, 930)	–	–
ART naive	–	701 (564, 813)	–	–
Viremic control	–	783 (525, 920)	–	–
Elite control	–	940 (821, 1009)	–	–
Lymphocytes (× 10 × 9/L)^a^		2 (1.7, 2.5)	2 (1.6, 2.5)	2.1 (1.8, 2.3)		0.438
Leukocytes (× 10 × 9/L)^a^		5.8 (4.8, 7.2)	5.6 (4.8, 6.7)	7.1 (5.2, 8.4)		0.011
HIV-1 RNA (copies/mL)^a^	Late presenters	–	178,500 (61,880, 340,300)	–		–
Discordant	–	< 40 (< 40, < 40)	–	–
Concordant	–	< 40 (< 40, < 40)	–	–
Early-treated	–	< 40 (< 40, < 40)	–	–
ART naive	–	13,900 (6867, 43,410)	–	–
Viremic control	–	794 (243, 1360)	–	–
Elite control	–	< 40 (< 40, < 40)	–	–

HTS, heterosexual; MSM, men who have sex with men; PWID, people who inject drugs; ATB, antibiotic; BCN Met, Barcelona Metropolitan Area; na, not available.

a Median (IQR), p-values for continuous and discrete variables were calculated with the Wilcoxon rank sum and Fisher's tests, respectively.

**Table 2:** Baseline chacteristics of subjects in the Stockholm validation dataset (STK).

		Full dataset	HIV-1 positive	HIV-1 negative	p-Value
No. of subjects		84	77	7	
Age (years)		40 (32, 48)	38 (32, 49)	44 (38, 47)	0.615
Gender	Male	51 (60.7%)	46 (59.7%)	5 (71.4%)	0.699
Female	33 (39.3%)	31 (40.3%)	2 (28.6%)
Risk group	HTS	55 (66.5%)	48 (62.3%)	7 (100%)	0.214
MSM	19 (22.6%)	19 (24.7%)	0
PWID	10 (11.9%)	10 (13.0%)	0
Ethnicity	Asian	2 (2.4%)	2 (2.6%)	0	1
Black	28 (33.3%)	26 (33.8%)	2 (28.6%)
Caucasian	52 (61.9%)	47 (61.0%)	5 (71.4%)
Hispanic–Latino	2 (2.4%)	2 (2.6%)	0
Country of origin	Sweden	39 (46.4%)	34 (43.4%)	5 (71.4%)	0.069
Kenya	5 (5.9%)	5 (6.5%)	0
Finland	4 (4.8%)	4 (5.2%)	0
Ethiopia	3 (3.6%)	1 (1.3%)	2 (28.6%)
Eritrea	3 (3.6%)	3 (3.9%)	0
Nigeria	3 (3.6%)	3 (3.9%)	0
Uganda	3 (3.6%)	3 (3.9%)	0
Other	24 (28.5%)	24 (31.2%)	0
CD4 + T-cell count (cells/mm^3^)^a^		–	480 (380, 630)	–	–
CD4 + T-cell count (%)^a^		–	26 (20, 32)	–	–
CD8 + T-cell count (cells/mm^3^)^a^		–	970 (660, 1290)	–	–
CD8 + T-cell count (%)^a^		–	51 (44, 59)	–	–
HIV-1 RNA copies/mL^a^		–	19,100 (1590, 69,900)	–	–

a Median (IQR), p-values for continuous and discrete variables were calculated with the Wilcoxon rank sum and Fisher's tests, respectively.
