# Supplementary material for: Gut Microbiota Linked to Sexual Preference and HIV Infection
Source: eBioMedicine. 2016 Jan 28;5:135–46. doi: 10.1016/j.ebiom.2016.01.032 (PMC4816837; doi:10.1016/j.ebiom.2016.01.032)
Supplement: Supplementary file 1 — Supplementary material [file mmc1.pdf]

Supplementary Materials

Gut microbiota linked to sexual preference and HIV infection

Marc Noguera-Julian[1,2,3]†, Muntsa Rocafort[1,3]†, Yolanda Guillén[1,3], Javier Rivera[1,2], Maria Casadellà[1,3], Piotr Nowak[4], Falk Hildebrand[5], Georg Zeller[5], Mariona Parera[1], Rocío Bellido[1], Cristina Rodríguez[1], Jorge Carrillo[1,3,7], Beatriz Mothe[1,2,3,6], Josep Coll[1,6], Isabel Bravo[6], Carla Estany[6], Cristina Herrero[6], Jorge Saz[8], Guillem Sirera[6], Ariadna Torrela[9], Jordi Navarro[9], Manel Crespo[9], Christian Brander[1,2,3,10], Eugènia Negredo[2,3,6], Julià Blanco[1,2,3], Francisco Guarner[11], Maria Luz Calle[2], Peer Bork[5,12,13], Anders Sönnerborg[4], Bonaventura Clotet[1,2,3,6], Roger Paredes*[1,2,3,6]

[1]irsiCaixa AIDS Research Institute, Ctra de Canyet s/n, 08916 Badalona, Catalonia, Spain.
[2]Universitat de Vic-Universitat Central de Catalunya, C. Sagrada Família 7, 08500 Vic, Catalonia, Spain.
[3]Universitat Autònoma de Barcelona, 08193 Bellaterra, Catalonia, Spain.
[4]Department of Medicine, Unit of Infectious Diseases, Karolinska University Hospital, Karolinska Institutet, Huddinge 141, 86 Stockholm, Sweden.
[5]Structural and Computational Biology, European Molecular Biology Laboratory, Meyerhofstrasse 1, 69117 Heidelberg, Germany.
[6]HIV Unit & Lluita contra la SIDA Foundation, Hospital Universitari Germans Trias i Pujol, Ctra de Canyet s/n, 08916 Badalona, Catalonia, Spain.
[7]ISGLOBAL, Carrer Rosselló, 132, 08036 Barcelona, Catalonia, Spain.
[8]BCN Checkpoint, Carrer del Comte Borrell, 164, 08015 Barcelona, Catalonia, Spain.
[9]Infectious Diseases Unit, Hospital Universitari Vall d'Hebrón, Passeig de la Vall d'Hebrón, 119-129, 08035 Barcelona, Catalonia, Spain.
[10]Institució Catalana de Recerca i Estudis Avançats (ICREA), Barcelona, Catalonia, Spain.
[11]Digestive Diseases Department, Vall d'Hebrón Institute of Research, Hospital Universitari Vall d'Hebrón, Passeig de la Vall d'Hebrón, 119-129, 08035 Barcelona, Catalonia, Spain.
[12]Max-Delbrück-Centre for Molecular Medicine, Robert-Rössle-Str. 10, 13092 Berlin, Germany.
[13]Molecular Medicine Partnership Unit, EMBL, Meyerhofstrasse 1, 69117 Heidelberg, Germany.

† Contributed equally to this work

*** Corresponding author:**
Dr. Roger Paredes, HIV Unit and irsiCaixa AIDS Research Institute, Ctra de Canyet s/n, 08916 Badalona, Catalonia, Spain. Tel: + 34 93 465 6374 (Ext: 162) email: rparedes@irsicaixa.es

Supplementary Methods

HIV phenotype definitions

To ensure that the study included a representative spectrum of different HIV-1 phenotypes, HIV-infected subjects had to belong to one of the following groups: (a) elite controllers, defined as subjects with HIV-1 RNA <50 copies/mL during at least 2 years in the absence of ART; (b) viremic controllers, with HIV-1 RNA between 50 - 2000 copies/mL during at least 2 years in the absence of ART; (c) ART naïve, with HIV-1 RNA >10,000 copies/mL, nadir CD4+ T-cell counts>500 cells/mm^3 and no ART exposure; (d) late presenters, with CD4+ T-cell counts < 200 cells/mm^3 at diagnosis and no ART exposure; (e) immune discordant, maintaining HIV-1 RNA <50 copies/mL and CD4+ T-cell counts <300 cells/mm^3 during at least 2 years under ART; (f) immune concordant: maintaining HIV-1 RNA levels <50 copies/mL and achieving CD4+ T-cell counts >500 cells/mm^3 during at least 2 years, and (g) early treated, defined as HIV-1-infected subjects who initiated ART during the first 6 months after the infection, achieving HIV-1 RNA levels <50 copies/mL during at least 3 months and with no HIV-1 RNA blips after achieving HIV-1 RNA < 50 copies/mL.

Fecal DNA extraction, amplicon generation and sequencing

DNA extraction was performed using the PowerSoil DNA Extraction Kit (MO BIO Laboratories, Carlsbad, CA, US). To amplify the variable region V3-V4 from the 16S rRNA gene (amplicon size expected ~460 bp), we used the primer pair described in the MiSeq rRNA Amplicon Sequencing protocol which already have the Illumina adapter overhang nucleotide sequences added to the 16S rRNA V3-V4 specific-primers, i.e.: 16S_F 5'-(TCG TCG GCA GCG TCA GAT GTG TAT AAG AGA CAG ***CCT ACG GGN GGC WGC AG)***-3' and 16S_R 5'-(GTC TCG TGG GCT CGG AGA TGT GTA TAA GAG ACA G***GA CTA CHV GGG TAT CTA ATC C)***-3'

Amplifications were performed in triplicate 25 µL reactions, each containing 2.5 µL of non-diluted DNA template, 12.5 µL of KAPA HiFi HotStart Ready Mix (containing KAPA HiFi HotStart DNA Polymerase, buffer, MgCl2, and dNTPs, KAPA Biosystems Inc., Wilmington, MA, USA), and 5 µL of each primer at 1 µM. Thermal cycling conditions consisted of an initial denaturation step (3 minutes at 95ºC), followed by 30 cycles of denaturation (30 seconds at 95ºC), annealing (30 seconds at 55ºC) and extension (30 seconds at 72ºC). These were followed by a final extension step of 10 minutes at 72ºC. Once the desired amplicon was confirmed in 1% agarose gel electrophoresis, all three replicates were pooled and stored at -30ºC until sequencing library preparation. Amplified DNA templates were cleaned-up for non-DNA molecules and Illumina sequencing adapters and dual indices were attached using Nextera XT Index Kit (Illumina, Inc.) followed by the corresponding PCR amplification program as described in the MiSeq 16S rRNA Amplicon Sequencing protocol. After a second round of cleanup, amplicons were quantified using Quant-iT™ PicoGreen® dsDNA Assay Kit (Invitrogen, Carlsbad, MA, USA) and diluted in equimolar concentrations (4 nM) for further pooling. Sequencing was performed on an Illumina MiSeq^{TM} platform

(Illumina, Inc.) according to the manufacturer's specifications to generate paired-end reads of 300 base-length in each direction.

Sequence Quality Control

The quality of MiSeq raw sequences was assessed using the FastQC software (Andrews 2010, http://www.bioinformatics.babraham.ac.uk/projects/fastqc/). Sequences were trimmed with Trimmommatic(Bolger et al., 2014) using a cutoff value of Q30 for both ends. Also, a minimum mean threshold of Q20 for 30bp-sliding window across sequences and a minimum read length of 250 bp was established.

16S rRNA Sequence Analysis

The Mothur software pipeline(Schloss et al., 2009) was used for the taxonomic classification of 16S rRNA sequences contained in each sample. We selected a random subset of 10,000 quality-filtered sequences per sample. In brief, Mothur performs various computational steps in order to bin sequences into operational taxonomic units (OTUs): Additional quality filtering was performed by allowing no ambiguous base calls and discarding reads out of the limits defined by primer design when aligned against SilvaDB(Quast et al., 2013; Yilmaz et al., 2014). Reference-based chimera filtering using the UCHIME(Edgar et al., 2011) algorithm and the Silva Gold alignment(Quast et al., 2013; Yilmaz et al., 2014), while the RDP classifier was used for taxonomical classification.(Wang et al., 2007) Sequences taxonomically classified as having mitochondrial, eukaryotic, chloroplast or unknown origin were disregarded. OTUs were clustered de-novo with a 97% similarity threshold using family level information to reduce the computational load. A representative sequence for each of the OTUs were extracted and a phylogenetic tree was created using FastTree2 software(Price et al., 2010) upon Mothur derived alignments in order to calculate phylogenetic UniFrac (weighted and unweighted) distance.(Price et al., 2010) To provide a technical validation of this procedure and confirm absence of user- or pipeline-dependent biases, the same analyses were repeated by a different research center using LotuS1.35(Hildebrand et al., 2014), using a maximum accumulated error of 0.75, a minimal average quality of 27 and truncating reads to 170 bp in short paired-end mode. For clustering, de novo chimera removal and denoising of OTUs UPARSE(Edgar, 2013) was used, also removing chimeric OTUs against the RDP reference database (http://drive5.com/uchime/rdp_gold.fa) with ref-based uchime(Edgar et al., 2011), merging reads with FLASH(Magoč and Salzberg, 2011) and assigning taxonomy of extended OTU seeds using the greengenes(McDonald et al., 2012) database and Blast. Clustal Omega(Sievers et al., 2011) multiple alignments were used with Fasttree2(Price et al., 2010) to construct a phylogenetic tree of de novo called OTUs. Downstream data analysis was performed with R software(Team, 2013) and a combination phyloseq,(McMurdie and Holmes, 2013) vegan,(Jari Oksanen, F. Guillaume Blanchet, Roeland Kindt, Pierre Legendre, Peter R. Minchin and Gavin L. Simpson, Peter Solymos, 2014) ade4(Dray and Dufour, 2007) R packages and ggplot2(Wickham, 2009) for data plotting.

Alpha and beta diversity analyses

To characterize alpha diversity in each dataset (BCN0, BCN1 and STK), species richness (Observed richness and the numeric richness estimators Chao1 and ACE) as well as diversity / evenness measurements (Shannon and Simpson indices) were calculated using Vegan(Jari Oksanen, F. Guillaume Blanchet, Roeland Kindt, Pierre Legendre, Peter R. Minchin and Gavin L. Simpson, Peter Solymos, 2014) and BiodiversityR R packages, correspondingly. OTUs present uniquely in one sample were filtered out to reduce noise. For each of the samples, a subset of 4000 counts was randomly selected using the *rrarefy* function from Vegan, as representative of the entire dataset.

The main study ordination analyses were performed using non-metric multidimensional scaling (NMDS) in a two dimensional space based on ecological distance matrices calculated by Bray-Curtis dissimilarities as implemented in R/phyloseq wrappers. The main findings were further confirmed using different ecological distances, as shown on Supplementary figures 6 to 8. Permutational analysis of variance using Bray-Curtis distances as implemented in *adonis* function from R/Vegan package was used to test the influence of different covariates on microbiome structure similarity measures.

To further explore if our datasets were structured in clusters, the Partitioning Around Medoids (PAM) algorithm,(Reynolds et al., 2006) as implemented in the R/cluster(Maechler, M., Rousseeuw, P., Struyf, A., Hubert, M., Hornik, 2015) package, was used considering Bray-Curtis distances between microbiota samples. Silhouette coefficients were used to assess the goodness-of-fit of the different possible partitions of each dataset. Two clusters were suggested in all datasets, which were labeled *Bacteroides* or *Prevotella* according to the relative abundance of these two genus in each of them.

Finally, to evaluate the concordance between the NMDS coordinate matrices of the BCN0 and BCN1 datasets we performed a Procrustes analysis(Gower, 1975) and used a Procrustean randomization test (PROTEST)(Jackson, 1995) to test for statistical significance.

Genus abundance

To assess genus abundance, OTU counts were collapsed to the bacterial genus level and genus proportions were calculated for each sample. The differential genus abundance by HIV-1 risk group (i.e., MSM, HTS and PWID) were compared using the Kruskal-Wallis and Dunn's post-hoc test when required. Only values achieving statistical significance after Benjamini-Hochberg correction(Hochberg and Benjamini, 1990) ($p<0.05$) were considered. In addition, a detailed description of the genus composition of each Bacteroides and Prevotella cluster was graphically summarized using word clouds implemented in the *wordcloud* R package.

To further identify patterns of genus distribution across the fecal microbiota samples in the different datasets, and their possible association with HIV-1 serostatus (HIV-1-infected vs. non-infected), HIV transmission group (MSM vs. non-MSM) and microbiota cluster (*Bacteroides* vs. *Prevotella*), we

represented the genus composition of each individual in consecutive bar plots ordered by the subject's coordinate in the first NMDS axis (NMDS1). The former covariates were represented on top of each bar plot using a color indicator.

Finally, we used the LEfSe algorithm(Segata et al., 2011) to describe which genus were significantly enriched in MSM vs. Non-MSM individuals consistently across HIV-1 serostatus (class: sexual orientation, subclass: HIV-1 status) and viceversa (class: HIV-1 status, subclass: sexual orientation). Comparisons were done in the Barcelona (BCN0) and Stockholm (STK) cohorts using the more stringent LEfSe criterion, i.e.: significant taxa had to differ between every pair of class values.

Microbiota classifier by HIV transmission group and HIV status

The observation of differences in genus abundance by HIV transmission group in the aforementioned comparisons prompted us to investigate if taxonomic microbial community composition could be associated with HIV transmission group (MSM vs. non-MSM) and, separately, with HIV-1 serostatus (HIV-1-positive vs. HIV-1-negative) using linear classification models. The statistical procedure (SIAMCAT) used in this manuscript was equivalent to one previously developed, tested and validated to classify risk of colorectal cancer based on fecal metagenomic biomarkers(Zeller et al., 2014) and is available online at siamcat.embl.de. To avoid confounding of the microbial HIV-1 classifier by HIV transmission group, it was only based on MSM samples.

In brief, the model used a LASSO logistic regression classifier(Tibshirani, 1994) (implemented in LIBLINEAR(Fan et al., 2008)), which generates a parsimonious classification model that only includes few features out of a potentially very large set. Together with the linearity of the model, this facilitates interpretation and marker extraction. Realistic estimates of its generalization error were obtained using cross-validation.

The SIAMCAT pipeline included the following steps: (a) Unsupervised feature abundance filtering to remove taxa whose relative abundance did not exceeded 0.001 in any sample; (b) Log-transform of the data and subsequent standardization of features (by centering to mean 0 and dividing by each features standard deviation to which we added the 10th percentile of standard deviations across all features); (c) Partitioning data for tenfold stratified cross-validation with resampling of dataset partitions ten times to obtain more stable accuracy estimates; (d) Fitting a LASSO model on the training data of each cross-validation fold: The LASSO hyperparameter was optimized for each model in a nested fivefold cross-validation on the training subset using the area under the ROC curve as model selection criterion and also enforcing at least five nonzero coefficients; (e) application of the trained LASSO models to obtain the corresponding cross-validation test predictions. Due to the resampled cross-validation, there are several test predictions for each test example. Hence, to get a single prediction score per example, all test predictions were averaged (from 100 models); (f) Model evaluation using ROC analysis from mean test prediction scores with 95% confidence intervals computed using the pROC R package, and (g) Model

interpretation and marker extraction: Bacterial species with potential as biomarkers were extracted as nonzero coefficients from all 100 LASSO models; those with a nonzero coefficient in at least 50% of the LASSO models were displayed in the order of their mean percentage of total absolute coefficient weight across all models. Bar lengths in Figure 3 directly correspond to mean log-odds ratios across LASSO models.

Functional profiling of the microbiota

Entire 16S rRNA BCN0 sequence dataset was independently analyzed using Mothur (Schloss et al., 2009) in combination with the GreenGenes database (McDonald et al., 2012). Additional quality filtering was performed by allowing no ambiguous basecalls and discarding reads outside the limits defined by the primer design when aligned against the GreenGenesDB. Chimeras were removed based on reference-based filtering using the uchime(Edgar et al., 2011) algorithm and the GreenGeenes alignment. The latter was also used for taxonomical classification using the RDP classifier. To ascertain the taxonomic composition from the whole data set, and not from a data subset, at a feasible computational load, and since de-novo OTU discovery was not of interest in this case, we followed the phylotype approach implemented in mothur, which allows for taxonomical classification of sequences, obtaining a phylotype table. The PICRUSt (Langille et al., 2013) software was used to infer the functional content of phylotypes. PICRUSt links 16S rRNA taxonomical identifiers and bacterial gene content, but only provides support for GreenGenes database identifiers. Briefly, the phylotype table was normalized by dividing the abundance of each organism by its predicted 16S rRNA copy number. To characterize the gene content, normalized phylotype abundances were multiplied by the respective set of gene abundances (represented by KEGG(Kanehisa and Goto, 2000) identifiers) estimated for each taxon. The gene content table obtained was then used to analyze the metabolic pathways represented at different KEGG level categories structured in three hierarchical levels using MinPath(Ye and Doak, 2009) implemented in the HUMAnN pipeline(Abubucker et al., 2012). Finally we identified the putative biologically relevant pathways that characterized the different bacterial clusters and/or HIV status by applying the LEfSe algorithm.(Segata et al., 2011)

Nutritional assessment and nutrient data analysis

The nutritional assessment was only performed in Barcelona. In a first visit, the HIV Unit dietitian provided two different validated questionnaires to the study participant and explained him/her how to complete them.

a. The first questionnaire was a standardized **prospective dietary nutrient survey** consisting on recording, as precisely as possible, everything that the patient ate or drank during 3 to 5 consecutive days, including al least one weekend day. Particular emphasis was put on recording the amounts of dietary complements like sugar, salt, sauces, etc. or the amount of oil used for

cooking or dressing, for example. The collected information was translated into numerical estimates of energy (kcal), and grams and percentage of the total energy intake contributed by proteins, total lipids, saturated, monounsaturated and polyunsaturated fat, total carbohydrates, sugars and digestive polysaccharides, as well as grams of total dietary fiber, vitamins and minerals. Such transformation was done using non-commercial software (*Programa de Càlcul Nutricional - PCN Pro, version 1.0, compilation 32, authors: David Cantós López, Andreu Farran Codina and Imma Palma Linares)* developed by the University of Barcelona. This program implements the nutrient composition tables developed by the *Centre d'Ensenyament de Nutrició Humana i Dietètica, CESNID (Tablas de composición de alimentos del CESNID, ISBN 84-486-0590-X),* which are the validated reference tables for nutritional composition used by all dietitians and nutritionists in Catalonia.

b. A second questionnaire was a **recall of food portions** taken per week, on average, over the last year. For example, how many glasses of milk the subject drinks per week: 1 per day, 1 every 2-3 days, etc. To a great extent, this questionnaire is equivalent to the one used by the Health Professionals Follow-up Study of the Harvard School of Public Health (http://www.hpfstudy.org)(Wu et al., 2011), but has been adapted to include local dietary products and exclude items that are almost not consumed at all in our environment (e.g. peanut butter, etc).

After completion of both questionnaires, the study subject reviewed them with the study dietitian. This allowed clarifying questions, identifying errors in completion and reaching a final agreement on diet consumption.

Energy intake was compared by microbiome clusters and by HIV transmission group using a Mann-Whitney U test. Individual nutritional data was then standardized by energy intake by fitting a linear model and taking residuals as new nutritional values (mean= 0 and SD=1).(Willett et al., 1997) This ensured that amounts of nutrients were comparable between subjects with different total energy intake.

A Dirichlet-multinomial distribution was assumed for genus counts to account for data overdispersion, as suggested previously(la Rosa et al., 2012). To identify which nutrients are more closely linked to each genus, Chen and Li recently suggested using the Dirichlet-Multinomial distribution to implement a penalized logistic regression model considering a number of parameters related with the mean and variance of the distribution of genus.(Chen and Li, 2013) Such Dirichlet multinomial regression was used to identify the strongest relationships between nutritional intake and genus abundance and variance in our dataset by applying strong penalties to weak associations using a penalized likelihood approach. Equivalent analyses were performed for prospectively estimated nutrient amounts and recalled food portions.

To obtain an idea of the dietary magnitude of the associations observed, the amounts of nutrients highlighted by the Dirichlet-Multinomial regression model where plotted against the first principal component (PCA1) of the microbiota distribution in Barcelona, as well as against the abundance of the genus *Bacteroides* and *Prevotella* in each microbiome. A linear regression model (Pearson regression) was fitted to analyze the linear correlation between such covariates and the standardized amount of nutrients and food portions using r-quare and raw and Benjamini-Hochberg-adjusted p-values.

In addition, Wilcoxon Mann–Whitney non-parametric test was used to evaluate differences in overall nutritional intake (nutrients and portions) between groups; p-values were corrected for multiple comparisons using the Benjamini-Hochberg method.

Moreover, to assess the global relationship between nutrients / food portions and the overall composition of each microbiome cluster (*Bacteroides* vs. *Prevotella*), as in(Wu et al., 2011), we calculated Spearman correlations between normalised amounts of nutrients and Bray-Curtis distance to the furthest subject in the opposite cluster. Negative correlations thus implied increased amounts of nutrient with shorter distance to each cluster, i.e.: higher consumption of that particular nutrient within the cluster, being displayed in red in a correlation heatmap. Strength of associations was described using r-square and raw and Benjamini-Hochberg-adjusted p-values. A Permanova test was used to assess overall differences in nutrient consumption between clusters.

The relative consumption of nutrients and food portions by HIV transmission group (MSM vs non-MSM) and microbiome cluster (*Bacteroides* vs. *Prevotella*) was assessed using bootstrapping to build 95% confidence intervals for the mean difference between groups.

Finally, A LASSO logistic regression classifier was also implemented with microbiome cluster (*Bacteroides* vs. *Prevotella*),as dependent variable and nutrients as covariates within a 10-fold cross-validation process. The model obtained, the one with minimum deviance corresponding to the optimal penalization parameter lambda, was the null model: no covariate (nutrient) was selected in this multivariate analysis.

Soluble markers of enterocyte integrity, bacterial translocation and systemic inflammation

Plasma samples were collected and cryopreserved at -80ºC until quantification. Plasma levels markers of enterocyte damage (intestinal fatty acid-binding protein, IFABP), microbial translocation (soluble CD14 (sCD14) and Lipopolysaccharide binding protein (LBP)) and systemic inflammation (interleukin-6 (IL-6), C-reactive protein (CRP) and interferon-gamma-inducible protein-10 (IP-10)) were analyzed using commercial ELISA kits in accordance with manufacturer's instructions [IL-6: High sensitivity ELISA with signal amplification, BMS213HS, eBioscience (San Diego, CA); sCD14: 0014-27, Diaclone (Besançon,

France); IFABP: DuoSet ELISA development system, R&D systems (Minneapolis, MN); CRP: KHA0031, Invitrogen (Carlsbad, CA); IP-10: 1P10-09, Diaclone (Besançon, France); LBP: KA0448, Abnova (Taipei, Taiwan)]. Results were analyzed and plotted using Graphpad Prism (v.5.0a GraphPad Software, San Diego California USA). If plasma levels were above the upper detection limit, its concentration was calculated as the maximum quantifiable value, i.e. the highest concentration included in the standard curve.

Plasma levels of soluble markers were compared by *Bacteroides* or *Prevotella* cluster using a Mann-Whitney U test, as well as by HIV-1 phenotype and HIV-1 risk group using a Kruskal-Wallis test. For deeper analysis of pairwise comparisons in the latter two cases, a post-hoc Dunn Test was run and resulting p-values where adjusted by the Benjamini–Hochberg (Hochberg and Benjamini, 1990) method to correct for multiple comparisons. Moreover, associations between the frequency of each of the 10 most abundant bacterial genus (i.e., those with a mean frequency of at least 2% across samples) and each soluble marker were evaluated by Pearson correlation using raw and Benjamini-Hochberg adjusted p-values.

Community Involvement

The study concept, design, patient information and results were discussed with the irsiCaixa Community Advisory Committee (CAC), who provided input on these aspects as well as on the presentation and dissemination of study results. The CAC is formed by 14 volunteer members, who represent the plurality and the different aspects around HIV. These members belong to local and regional public institutions, NGOs, people participating in clinical trials, people living with HIV, men who have sex with men and sex workers. At present, the CAC is formed by members of *Àmbit Prevenció*, *Gais Positius*, the *Consorci Sanitari de Barcelona* (Public Health Agency of Barcelona), the *Federación de Entidades Latinoamericanas de Catalunya*, *Planeta Salud*, the Program for the Prevention and Assistance of AIDS (from the Health Department of Health of the Generalitat de Catalunya) and the *Projecte dels Noms-Hispanosida* organization, as well as volunteers participating in studies. The mission of this board is to provide researchers with a wider and complementary view of the impact, consequences and viability of the studies they carry out, including ethical, legal and social aspects. The CAC meetings are held four times a year and its main functions are to offer non-binding advice to researchers, to review the research projects and to monitor the impact that the studies may have in the different affected groups and fields. The CAC also aims to evaluate information and educational materials and facilitating the community access to the latest research results.

References

Abubucker, S., Segata, N., Goll, J., Schubert, A.M., Izard, J., Cantarel, B.L., Rodriguez-Mueller, B., Zucker, J., Thiagarajan, M., Henrissat, B., White, O., Kelley, S.T., Methé, B., Schloss, P.D., Gevers, D., Mitreva, M., Huttenhower, C., 2012. Metabolic Reconstruction for Metagenomic Data and Its Application to the Human Microbiome. PLoS Comput. Biol. 8, e1002358. doi:10.1371/journal.pcbi.1002358

Bolger, A.M., Lohse, M., Usadel, B., 2014. Trimmomatic: a flexible trimmer for Illumina sequence data. Bioinformatics 30, 2114–20. doi:10.1093/bioinformatics/btu170

Chen, J., Li, H., 2013. Variable selection for sparse dirichlet-multinomial regression with an application to microbiome data analysis. Ann. Appl. Stat. 7. doi:10.1214/12-AOAS592

Dray, S., Dufour, A.B., 2007. The ade4 package: implementing the duality diagram for ecologists. J. Stat. Softw. 22, 1–20.

Edgar, R.C., 2013. UPARSE: highly accurate OTU sequences from microbial amplicon reads. Nat. Methods 10, 996–8. doi:10.1038/nmeth.2604

Edgar, R.C., Haas, B.J., Clemente, J.C., Quince, C., Knight, R., 2011. UCHIME improves sensitivity and speed of chimera detection. Bioinformatics 27, 2194–200. doi:10.1093/bioinformatics/btr381

Fan, R., Chang, K., Hsieh, C., 2008. LIBLINEAR: A library for large linear classification. J. Mach. Learn. 9, 1871–1874. doi:10.1038/oby.2011.351

Gower, J.C., 1975. Generalized procrustes analysis. Psychometrika 40, 33–51. doi:10.1007/BF02291478

Hildebrand, F., Tadeo, R., Voigt, A.Y., Bork, P., Raes, J., 2014. LotuS: an efficient and user-friendly OTU processing pipeline. Microbiome 2, 1–7. doi:10.1186/2049-2618-2-30

Hochberg, Y., Benjamini, Y., 1990. More powerful procedures for multiple significance testing. Stat. Med. 9, 811–8.

Jackson, D.A., 1995. Protest - A Procrustean Randomization Test of Community Environment Concordance. Ecoscience 2, 297–303. doi:citeulike-article-id:9258086

Jari Oksanen, F. Guillaume Blanchet, Roeland Kindt, Pierre Legendre, Peter R. Minchin, R.B.O., Gavin L. Simpson, Peter Solymos, M.H.H.S. and H.W., 2014. vegan: Community Ecology Package.

Kanehisa, M., Goto, S., 2000. KEGG: kyoto encyclopedia of genes and genomes. Nucleic Acids Res. 28, 27–30.

la Rosa, P.S., Brooks, J.P., Deych, E., Boone, E.L., Edwards, D.J., Wang, Q., Sodergren, E., Weinstock, G., Shannon, W.D., 2012. Hypothesis Testing and Power Calculations for Taxonomic-Based Human Microbiome Data. PLoS One 7, 1–13. doi:10.1371/journal.pone.0052078

Langille, M.G.I., Zaneveld, J., Caporaso, J.G., McDonald, D., Knights, D., Reyes, J. a, Clemente, J.C., Burkepile, D.E., Vega Thurber, R.L., Knight, R., Beiko, R.G., Huttenhower, C., 2013. Predictive functional profiling of microbial communities using 16S rRNA marker gene sequences. Nat. Biotechnol. 31, 814–21. doi:10.1038/nbt.2676

Maechler, M., Rousseeuw, P., Struyf, A., Hubert, M., Hornik, K., 2015. cluster: Cluster Analysis Basics and Externsions.

Magoč, T., Salzberg, S.L., 2011. FLASH: fast length adjustment of short reads to improve genome assemblies. Bioinformatics 27, 2957–63. doi:10.1093/bioinformatics/btr507

McDonald, D., Price, M.N., Goodrich, J., Nawrocki, E.P., DeSantis, T.Z., Probst, A., Andersen, G.L.,

Knight, R., Hugenholtz, P., 2012. An improved Greengenes taxonomy with explicit ranks for ecological and evolutionary analyses of bacteria and archaea. ISME J. 6, 610–8. doi:10.1038/ismej.2011.139

McMurdie, P.J., Holmes, S., 2013. phyloseq: an R package for reproducible interactive analysis and graphics of microbiome census data. PLoS One 8, e61217. doi:10.1371/journal.pone.0061217

Price, M.N., Dehal, P.S., Arkin, A.P., 2010. FastTree 2--approximately maximum-likelihood trees for large alignments. PLoS One 5, e9490. doi:10.1371/journal.pone.0009490

Quast, C., Pruesse, E., Yilmaz, P., Gerken, J., Schweer, T., Yarza, P., Peplies, J., Glöckner, F.O., 2013. The SILVA ribosomal RNA gene database project: improved data processing and web-based tools. Nucleic Acids Res. 41, D590–6. doi:10.1093/nar/gks1219

Reynolds, A.P., Richards, G., de la Iglesia, B., Rayward-Smith, V.J., 2006. Clustering Rules: A Comparison of Partitioning and Hierarchical Clustering Algorithms. J. Math. Model. Algorithms 5, 475–504. doi:10.1007/s10852-005-9022-1

Schloss, P.D., Westcott, S.L., Ryabin, T., Hall, J.R., Hartmann, M., Hollister, E.B., Lesniewski, R.A., Oakley, B.B., Parks, D.H., Robinson, C.J., Sahl, J.W., Stres, B., Thallinger, G.G., Van Horn, D.J., Weber, C.F., 2009. Introducing mothur: open-source, platform-independent, community-supported software for describing and comparing microbial communities. Appl. Environ. Microbiol. 75, 7537–41. doi:10.1128/AEM.01541-09

Segata, N., Izard, J., Waldron, L., Gevers, D., Miropolsky, L., Garrett, W.S., Huttenhower, C., 2011. Metagenomic biomarker discovery and explanation. Genome Biol. 12, R60. doi:10.1186/gb-2011-12-6-r60

Sievers, F., Wilm, A., Dineen, D., Gibson, T.J., Karplus, K., Li, W., Lopez, R., McWilliam, H., Remmert, M., Söding, J., Thompson, J.D., Higgins, D.G., 2011. Fast, scalable generation of high-quality protein multiple sequence alignments using Clustal Omega. Mol. Syst. Biol. 7, 539. doi:10.1038/msb.2011.75

Team, R.C., 2013. R: A Language and Environment for Statistical Computing.

Tibshirani, R., 1994. Regression Selection and Shrinkage via the Lasso. J. R. Stat. Soc. B. doi:10.2307/2346178

Wang, Q., Garrity, G.M., Tiedje, J.M., Cole, J.R., 2007. Naive Bayesian classifier for rapid assignment of rRNA sequences into the new bacterial taxonomy. Appl. Environ. Microbiol. 73, 5261–7. doi:10.1128/AEM.00062-07

Wickham, H., 2009. ggplot2: elegant graphics for data analysis. Springer, New York.

Willett, W.C., Howe, G.R., Kushi, L.H., 1997. Adjustment for total energy intake in epidemiologic studies. Am. J. Clin. Nutr. 65, 1220S–1228S; discussion 1229S–1231S.

Wu, G.D., Chen, J., Hoffmann, C., Bittinger, K., Chen, Y., Keilbaugh, S.A., Bewtra, M., Knights, D., Walters, W.A., Knight, R., Sinha, R., Gilroy, E., Gupta, K., Baldassano, R., Nessel, L., Li, H., 2011. Linking Long-term Dietary Patterns with Gut Microbial Enterotypes. Science 334, 105–108.

Ye, Y., Doak, T.G., 2009. A parsimony approach to biological pathway reconstruction/inference for genomes and metagenomes. PLoS Comput. Biol. 5, e1000465. doi:10.1371/journal.pcbi.1000465

Yilmaz, P., Parfrey, L.W., Yarza, P., Gerken, J., Pruesse, E., Quast, C., Schweer, T., Peplies, J., Ludwig, W., Glöckner, F.O., 2014. The SILVA and “All-species Living Tree Project (LTP)” taxonomic frameworks. Nucleic Acids Res. 42, D643–8. doi:10.1093/nar/gkt1209

Zeller, G., Tap, J., Voigt, A.Y., Sunagawa, S., Kultima, J.R., Paul, I., Amiot, A., Böhm, J., Brunetti, F., Habermann, N., Hercog, R., Koch, M., Luciani, A., Mende, D.R., Schneider, M.A., Schrotz-king, P., Tournigand, C., Nhieu, J.T. Van, Yamada, T., Zimmermann, J., 2014. Potential of fecal microbiota for early-stage detection of colorectal cancer. Mol. Syst. Biol. 10, 1–18.

Supplementary Table 1. ADONIS test of factors explaining distance variations among human fecal microbiomes in the BCN0 dataset

Variable	Univariate			Multivariate*	
	R2	p-value		R2	p-value
**HIV Risk Group**	0.367	<0.001		**0.373**	**<0.001**
Gender	0.152	<0.001		0.008	0.407
Feces consistency	0.049	<0.001		0.009	0.335
Residency	0.044	0.014		0.008	0.421
Ethnicity	0.037	0.022		0.018	0.156
HIV serostatus	0.035	0.003		0.011	0.054
Altered Abdominal transit	0.031	0.023		0.005	0.238

* terms added sequentially (first to last)

Supplementary Table 2. Potential microbiota confounders in MSM vs. non-MSM. Barcelona test (BCN0) dataset

		All (n=156)	MSM (n=100)	Non-MSM (n=56)	P-value	
**Age (years)***		43 (35, 51)	38 (34, 46.3)	50 (42, 54)	<0.001	
**Ethnicity**	**Asiatic**	1 (0.6%)	0	1 (1.8%)	0.163	0.359
	**Caucasian**	124 (79.5%)	78 (78%)	46 (82.1%)		0.680
	**Hispanic-Latino**	28 (18%)	21 (21%)	7 (12.5%)		0.201
	**Others**	3 (1.9%)	1 (1%)	2 (3.6%)		0.293
**Residency**	**Barcelona**	51 (32.7%)	41 (41%)	10 (17.9%)	0.022	0.004
	**BCN Met**	56 (35.8%)	30 (30%)	26 (46.4%)		0.055
	**Outside BCN Met**	38 (24.4%)	22 (22%)	16 (28.6%)		0.437
	**na**	11 (7.1%)	7 (7%)	4 (7.1%)		1
**BMI (kg/m^2) ***		23.8 (22, 26)	24.3 (22.3, 26.2)	23.5 (20.9, 25.17)	0.053	
**Allergy**	**No**	122 (78.2%)	79 (79%)	43 (76.8%)	0.876	0.840
	**Yes**	30 (19.2%)	18 (18%)	12 (21.4%)		0.673
	**na**	4 (2.6%)	3 (3%)	1 (1.8%)		1
**ATB during the previous 3-6 mo**		35 (22.4%)	20 (20%)	15 (26.8%)	0.424	
**Fecal consistency**	**Hard**	56 (35.9%)	26 (26%)	30 (53.6%)	<0.001	<0.001
	**Soft**	91 (58.3%)	68 (68%)	23 (41.1%)		0.001
	**Liquid**	5 (3.2%)	2 (2%)	3 (5.3%)		0.363
	**na**	4 (2.6%)	4 (4%)	0		0.297
**Abdominal transit alterations**	**Yes**	23 (14.7%)	14 (14%)	9 (16%)	0.191	0.815
	**No**	127 (81.4%)	80 (80%)	47 (84%)		0.669
	**na**	6 (3.9%)	6 (6%)	0		0.088
**Defecation frequency (per day)**	**1**	88 (56.4%)	51 (51%)	37 (66.1%)	0.221	0.091
	**2**	47 (30.1%)	31 (31%)	16 (28.6%)		0.856
	**3**	12 (7.7%)	10 (10%)	2 (3.5%)		0.131
	**4**	5 (3.2%)	4 (4%)	1 (1.8%)		0.655
	**na**	4 (2.6%)	4 (4%)	0		0.297
**HBV co-infection**	**Positive**	19 (12.2%)	2 (2%)	17 (30.4%)	<0.001	<0.001
	**Negative**	112 (71.8%)	79 (79%)	33 (58.9%)		0.009
	**na**	25 (16%)	19 (19%)	6 (10.7%)		0.255
**HCV co-infection**	**Positive**	24 (15.4%)	6 (6%)	18 (32.1%)	<0.001	<0.001
	**Negative**	120 (76.9%)	88 (88%)	32 (57.1%)		<0.001
	**na**	12 (7.7%)	6 (6%)	6 (10.8%)		0.352
**Syphilis serology**	**Positive**	21 (13.5%)	18 (18%)	3 (5.4%)	0.056	0.029
	**Negative**	116 (74.3%)	72 (72%)	44 (78.6%)		0.446
	**na**	19 (12.2%)	10 (10%)	9 (16%)		0.311
**PCR *Chlamydia trachomatis***	**Positive**	9 (5.8%)	8 (8%)	1 (1.8%)	0.216	0.158
	**Negative**	115 (73.7%)	74 (74%)	41 (73.2%)		1
	**na**	32 (20.5%)	18 (18%)	14 (25%)		0.309
**PCR *Neisseria gonorrhoeae***	**Positive**	0	0	0	0.296	-
	**Negative**	125 (80.1%)	83 (83%)	42 (75%)		-
	**na**	31 (19.9%)	17 (17%)	14 (25%)		-
**PCR Human Papilloma Virus**	**Yes**	72 (46.2%)	49 (49%)	23 (41.1%)	0.229	0.403
	**No**	83 (53.2%)	51 (51%)	32 (57.1%)		0.506
	**na**	1 (0.6%)	0	1 (1.8%)		0.359
**Anal cytology**	**ASCUS**	22 (14.1%)	15 (15%)	7 (12.5%)	0.715	0.812
	**HSIL**	7 (4.5%)	5 (5%)	2 (3.6%)		1
	**LSIL**	30 (19.2%)	22 (22%)	8 (14.3%)		0.293
	**Normal**	80 (51.3%)	48 (48%)	32 (57.1%)		0.318
	**na**	17 (10.9%)	10 (10%)	7 (12.5%)		0.779

** median(IQR), p-values for continuous and discrete variables calculated with the Wilcoxon Rank Sum and Fisher's tests, respectively. MSM, Men who have sex with men; ATB, antibiotic: BCN Met, Barcelona Metropolitan Area; na, not available;*

Supplementary figure 1. Overall study strategy

The study was first performed in 156 subjects from a test dataset in Barcelona, Catalonia, (BCN at month 0, or BCN0), recruited by the irsiCaixa and Lluita contra la SIDA Foundations. Findings in BCN0 were internally validated by repeating the same analyses in the same cohort one month later (BCN month 1, or BCN1) in 110 individuals who provided samples at both timepoints. External validation of our findings was obtained by testing fecal samples from an independent cohort recruited by the Karolinska Institute in Stockholm, Sweden.

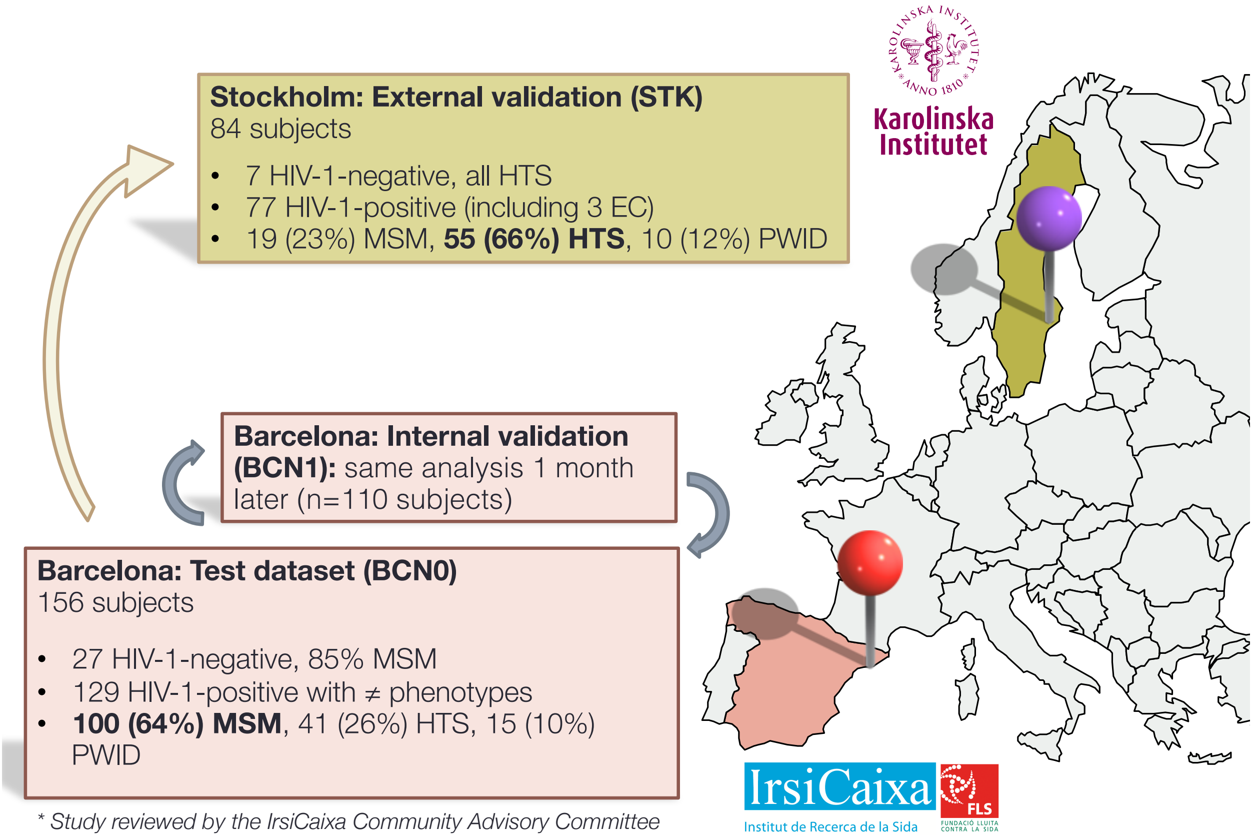

Supplementary figure 2. Internal validation of the influence of HIV transmission group and HIV-1 infection on human fecal microbiota alpha diversity: internal validation dataset from Barcelona, month 1 (BCN1)

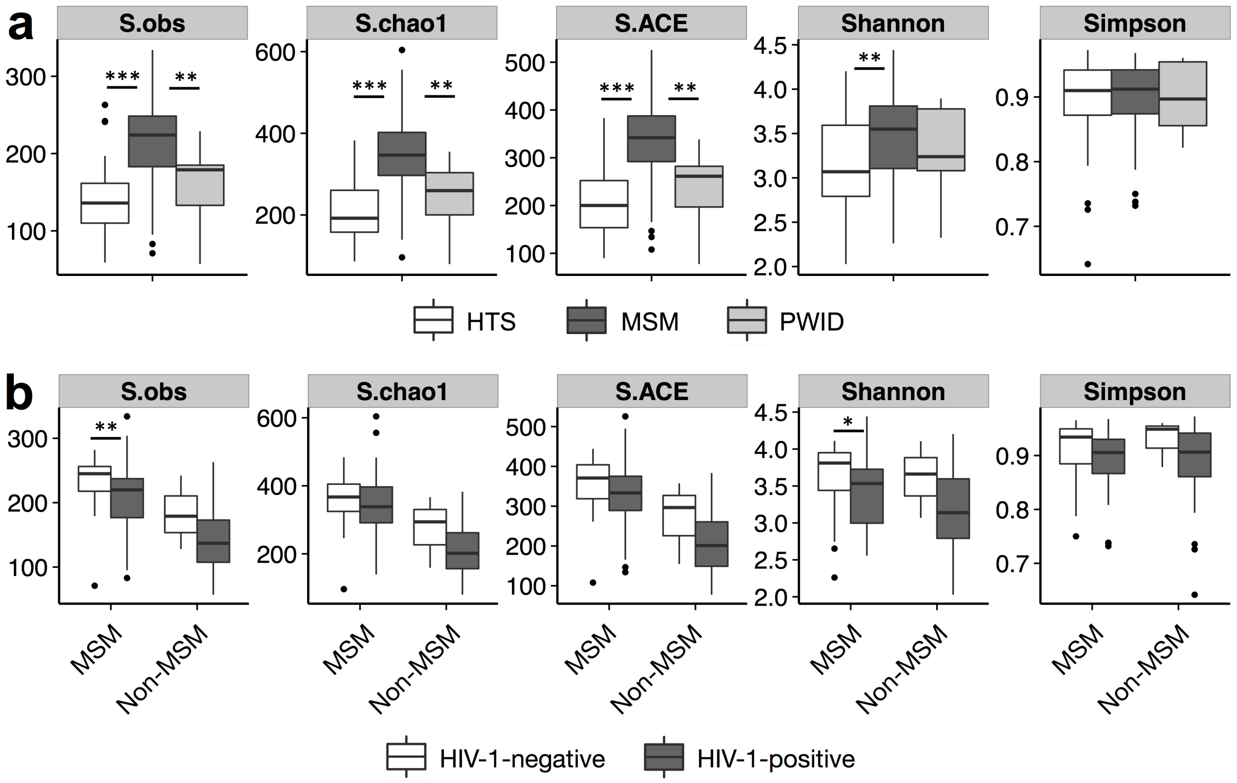

a, The highest richness and diversity in human fecal microbiota were observed in men who have sex with men (MSM). There were no differences between heterosexual subjects (HTS) and people who acquired HIV through intravenous drug use (PWID). Kruskal Wallis p-values were adjusted for multiple comparisons using Benjamini-Hochberg method. **b,** HIV-1 infection was associated with significant reductions in fecal human microbiome richness after stratifying for HIV transmission group. All alpha diversity findings were consistent in Barcelona (test cohort, month 0) (BCN) and Stockhom (STK). Note: “Simpson” refers to 1-Simpson index. The remaining ecological index names are self-explanatory. *p<0.1, **p<0.05, ***p<0.001

Supplementary figure 3. Word cloud plots of the genus composition of the *Bacteroides* and *Prevotella* clusters in Barcelona and Stockholm

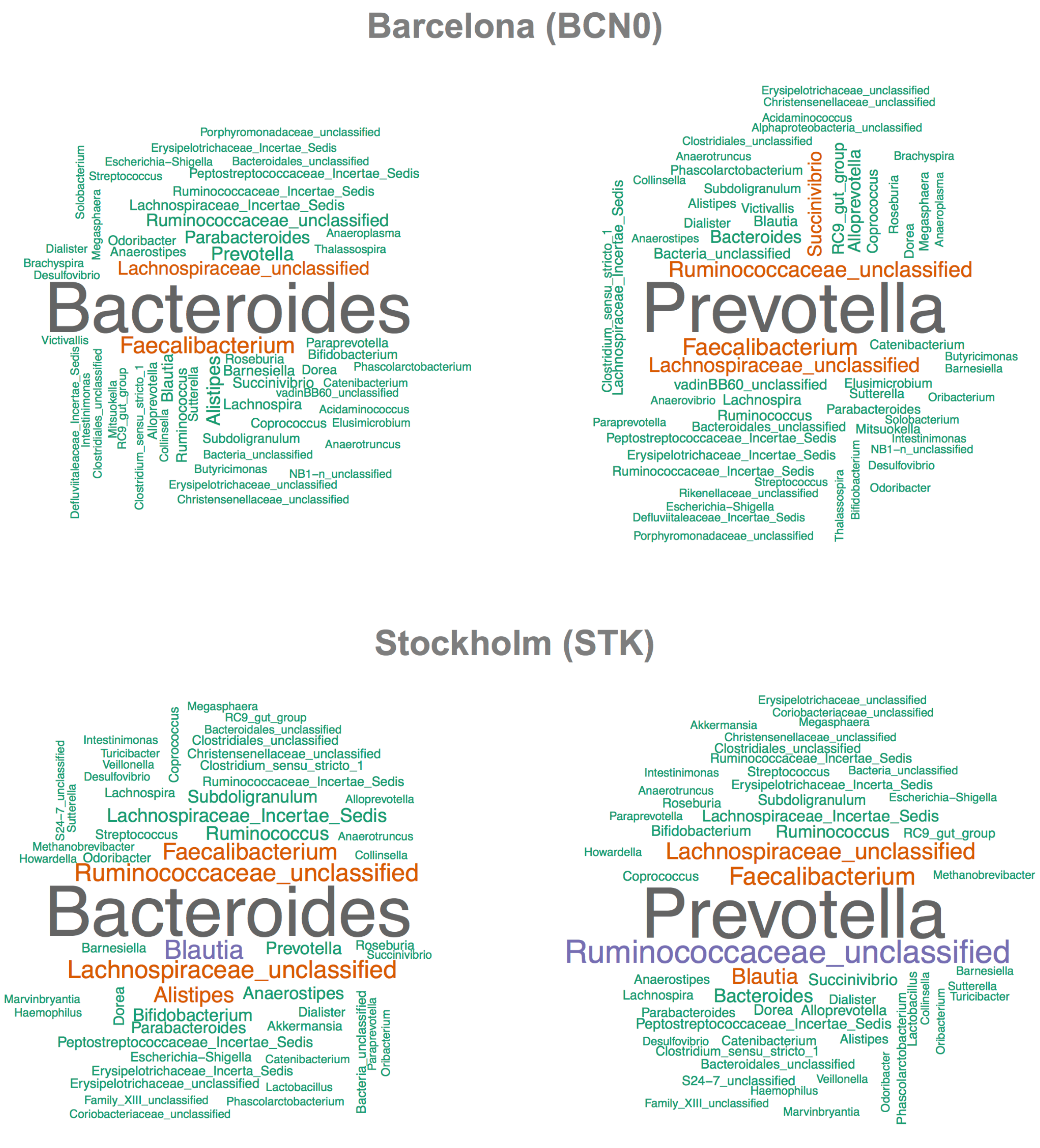

The *Bacteroides* clusters are shown on the left; the *Prevotella* clusters are shown on the right. The Barcelona dataset is the test dataset (BCN0). The relative abundance of each genus is proportional to the font size. Genus are also colored by abundance ranks, to ease the graphical interpretation. These word cloud plots show remarkable similarities in the genus composition of the fecal microbiota clusters in Barcelona and Stockholm.

Supplementary figure 4. Functional profile of the fecal microbiota clusters

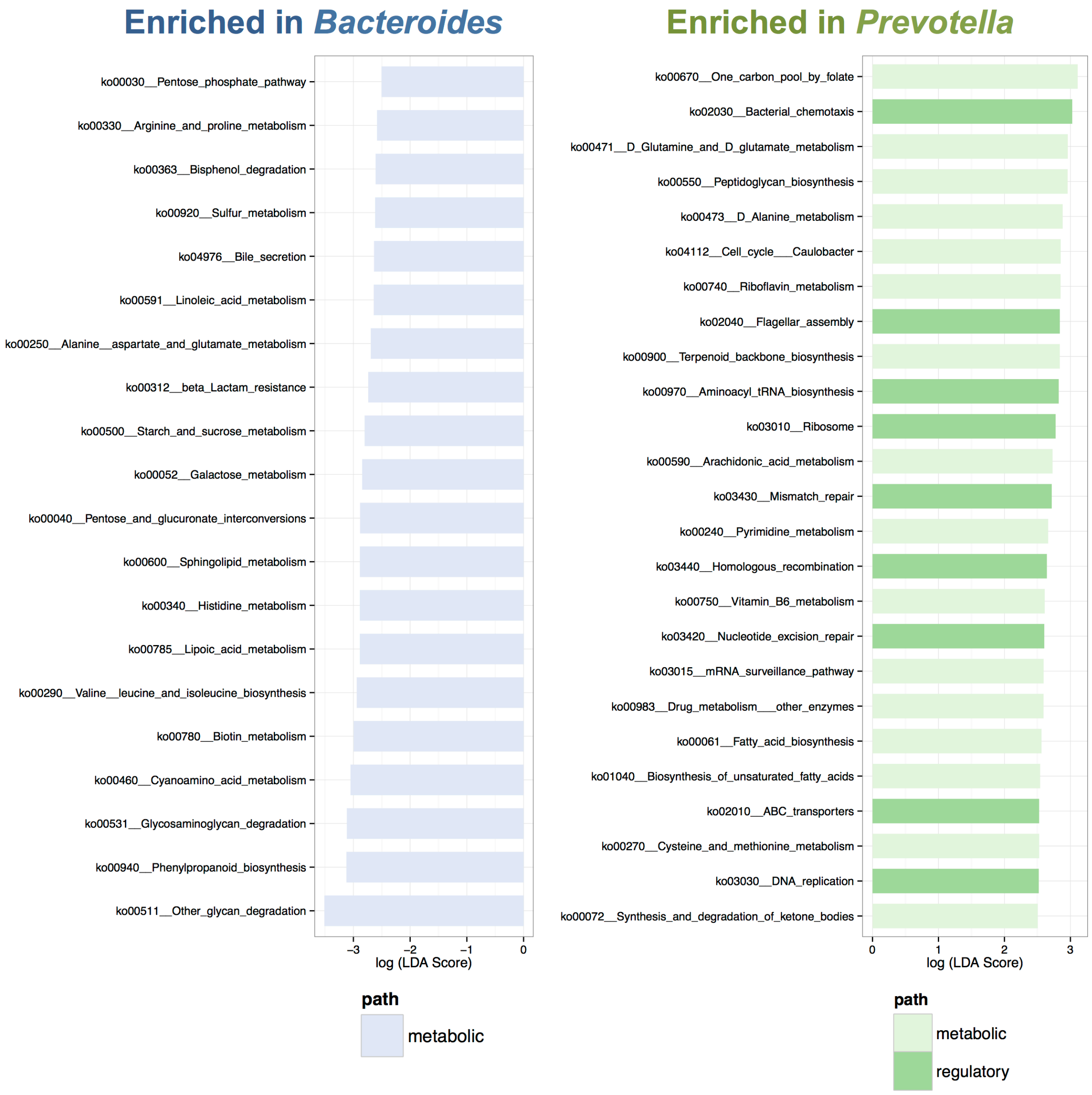

Metabolic and regulatory pathways enriched in the *Bacteroides* (blue) versus *Prevotella* (green) fecal microbiota clusters relative to each other, in the Barcelona test cohort (BCN0). Linear discriminative analysis (LDA) scores were obtained using LEfSe[1] on PICRUSt[2] and HUMAnN[3] data.

---

[1] *Segata, N. et al. Metagenomic biomarker discovery and explanation. Genome Biol. 12, R60 (2011)*

[2] *Langille, M. G. I. et al. Predictive functional profiling of microbial communities using 16S rRNA marker gene sequences. Nat. Biotechnol.* ***31,*** *814–21 (2013).*

[3] *Abubucker, S. et al. Metabolic Reconstruction for Metagenomic Data and Its Application to the Human Microbiome. PLoS Comput. Biol.* ***8,*** *e1002358 (2012).*

Supplementary figure 5. Metabolic pathway map of the *Bacteroides* and *Prevotella* clusters

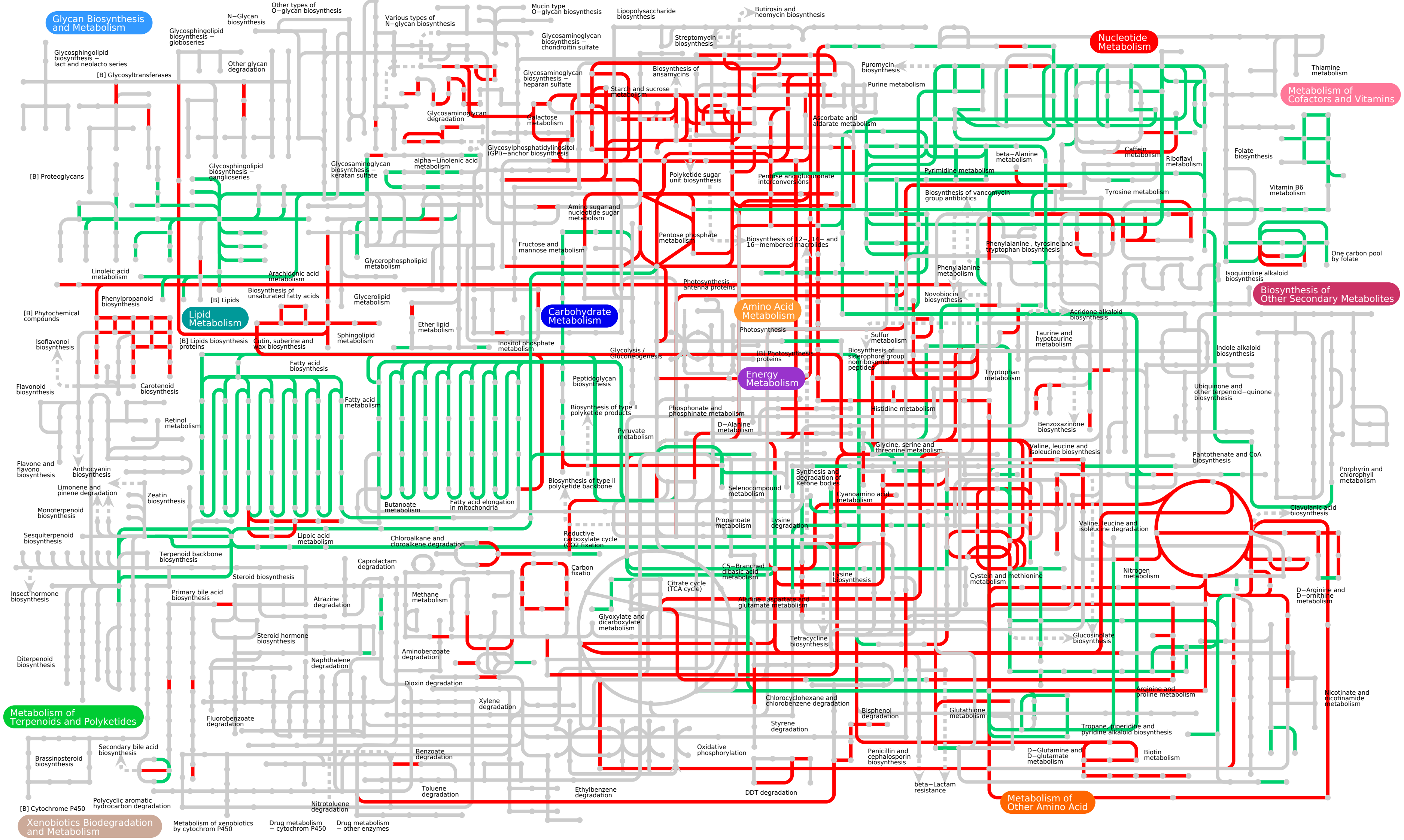

iPATH map of metabolic pathways of the *Bacteroides* (red) and *Prevotella (green)* clusters in the Barcelona test cohort (BCN0) obtained with PICRUSt

Supplementary figure 6. Ordination plots using different ecological distances: Barcelona test dataset (BCN0)

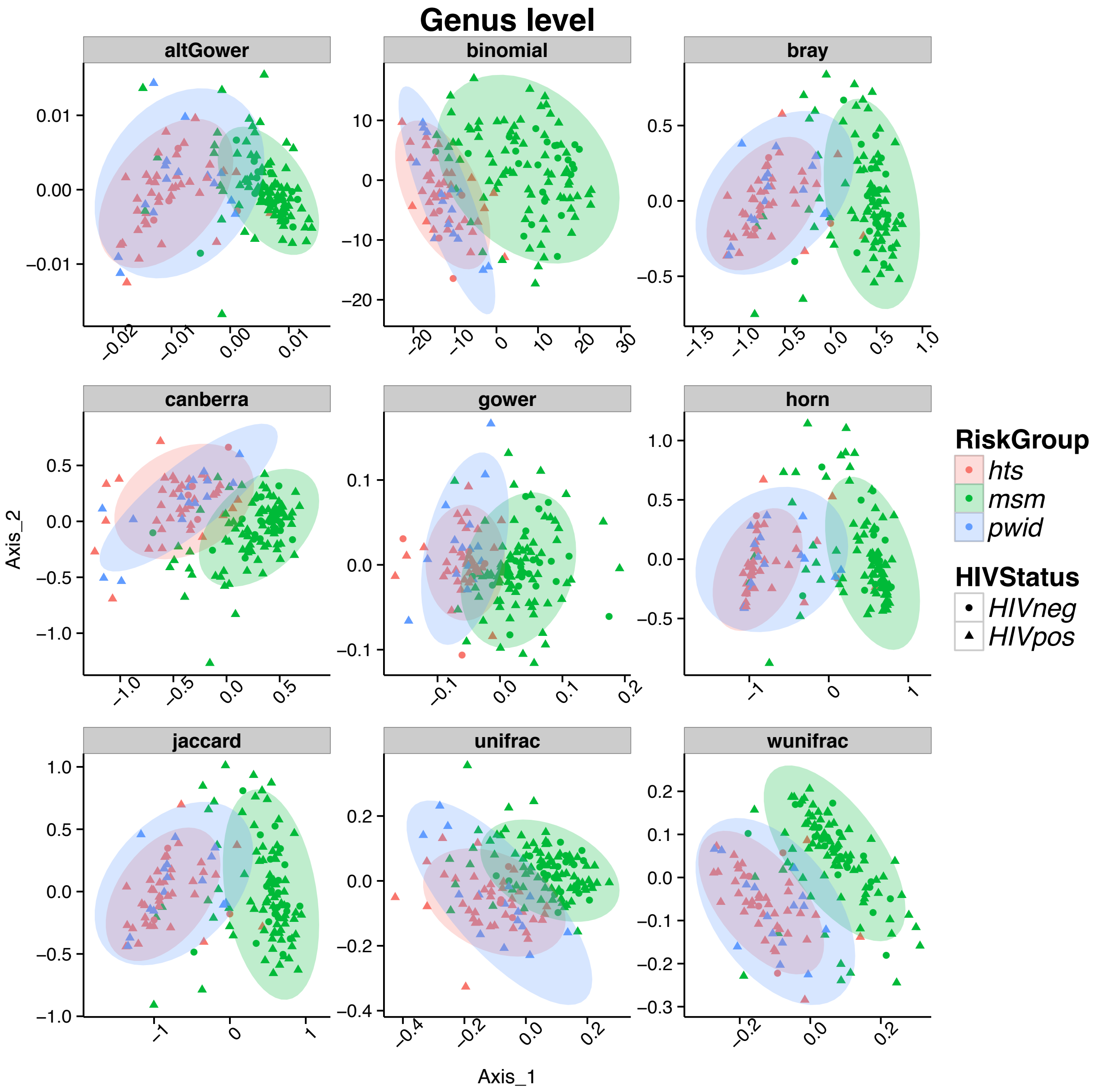

With different distance metrics, fecal microbiomes clustered mainly by risk group rather than by HIV-1 status. Plots showed that MSM represent a separated cluster whereas the non-MSM groups (HTS and PWID) largely overlap. Note: MSM: men who have sex with men; HTS, heterosexual subjects; PWID, people who inject or injected drugs; wunifrac, weighted unifrac. Ellipses include 95% of subjects within each cluster.

Supplementary figure 7. Ordination plots using different ecological distances: Barcelona internal validation dataset (BCN1)

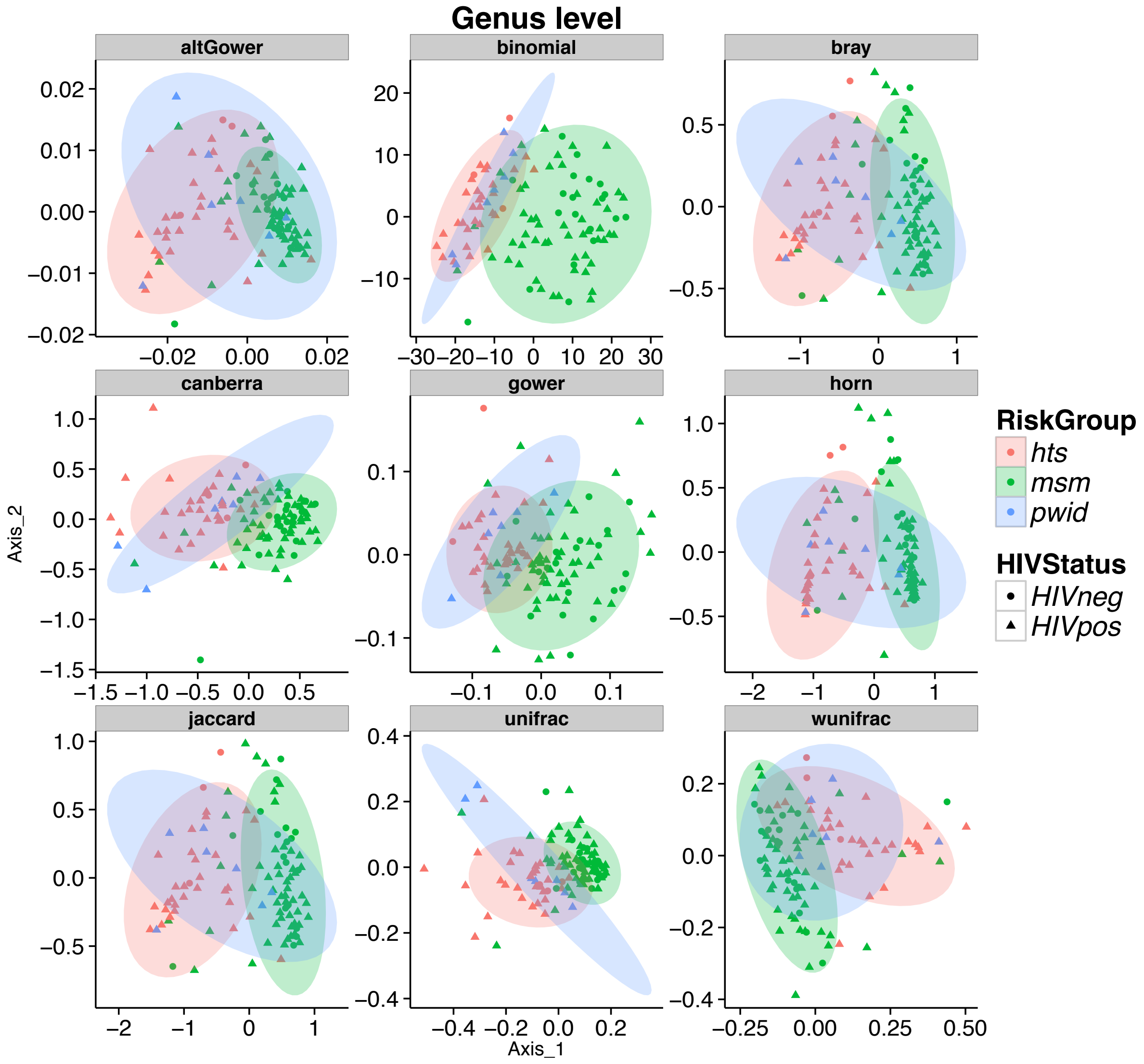

With different distance metrics, fecal microbiomes clustered mainly by risk group rather than by HIV-1 status. Plots showed that MSM represent a separated cluster whereas the non-MSM groups (HTS and PWID) largely overlap. Note: MSM: men who have sex with men; HTS, heterosexual subjects; PWID, people who inject or injected drugs; wunifrac, weighted unifrac. Ellipses include 95% of subjects within each cluster.

Supplementary figure 8. Ordination plots using different ecological distances: Stockholm (STK) external validation dataset.

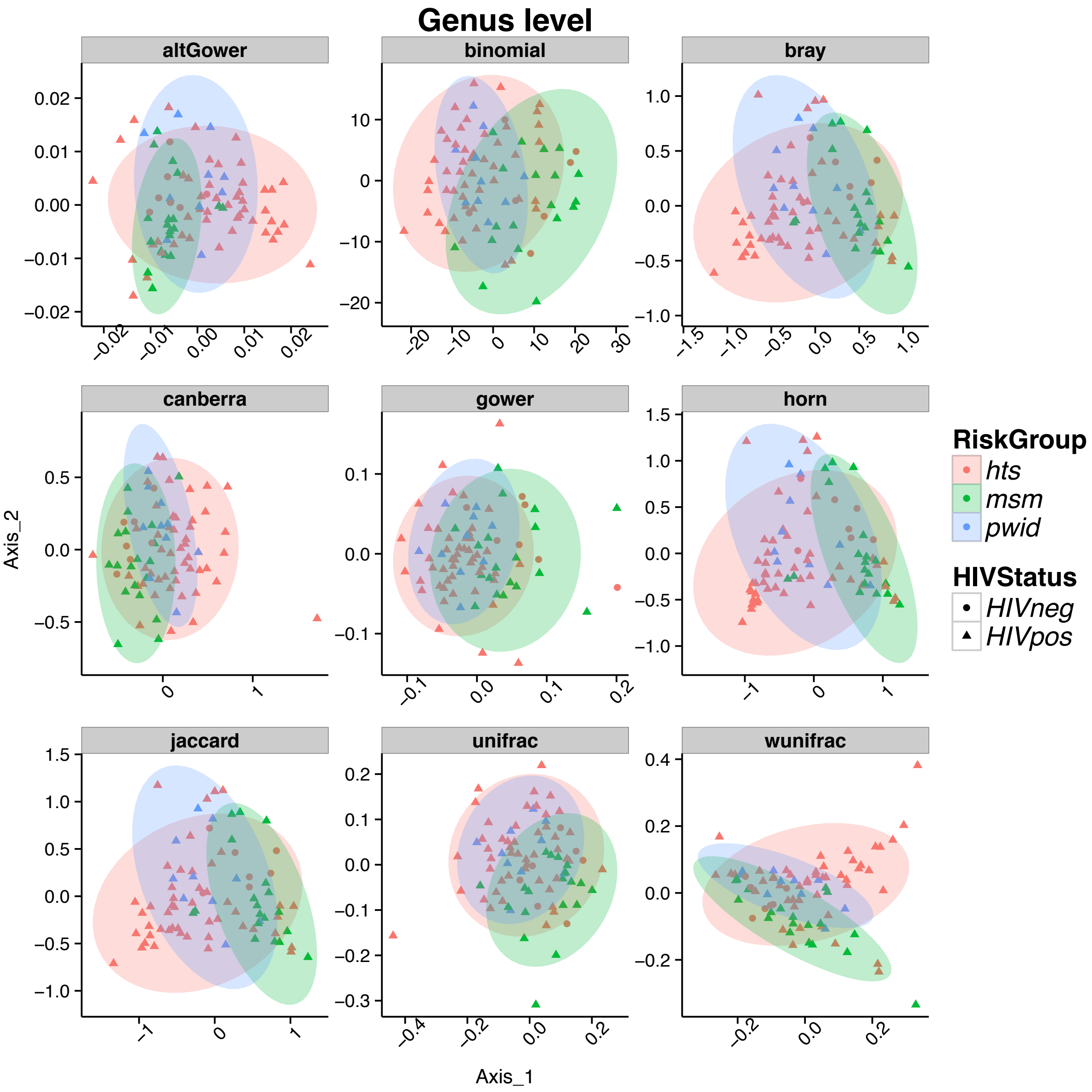

With different distance metrics, fecal microbiomes clustered mainly by risk group rather than by HIV-1 status. Note: MSM: men who have sex with men; HTS, heterosexual subjects; PWID, people who inject or injected drugs; wunifrac, weighted unifrac. Ellipses include 95% of subjects within each cluster.

Supplementary figure 9. Concordance between the Barcelona test (BCN0) and internal validation (BCN1) datasets.

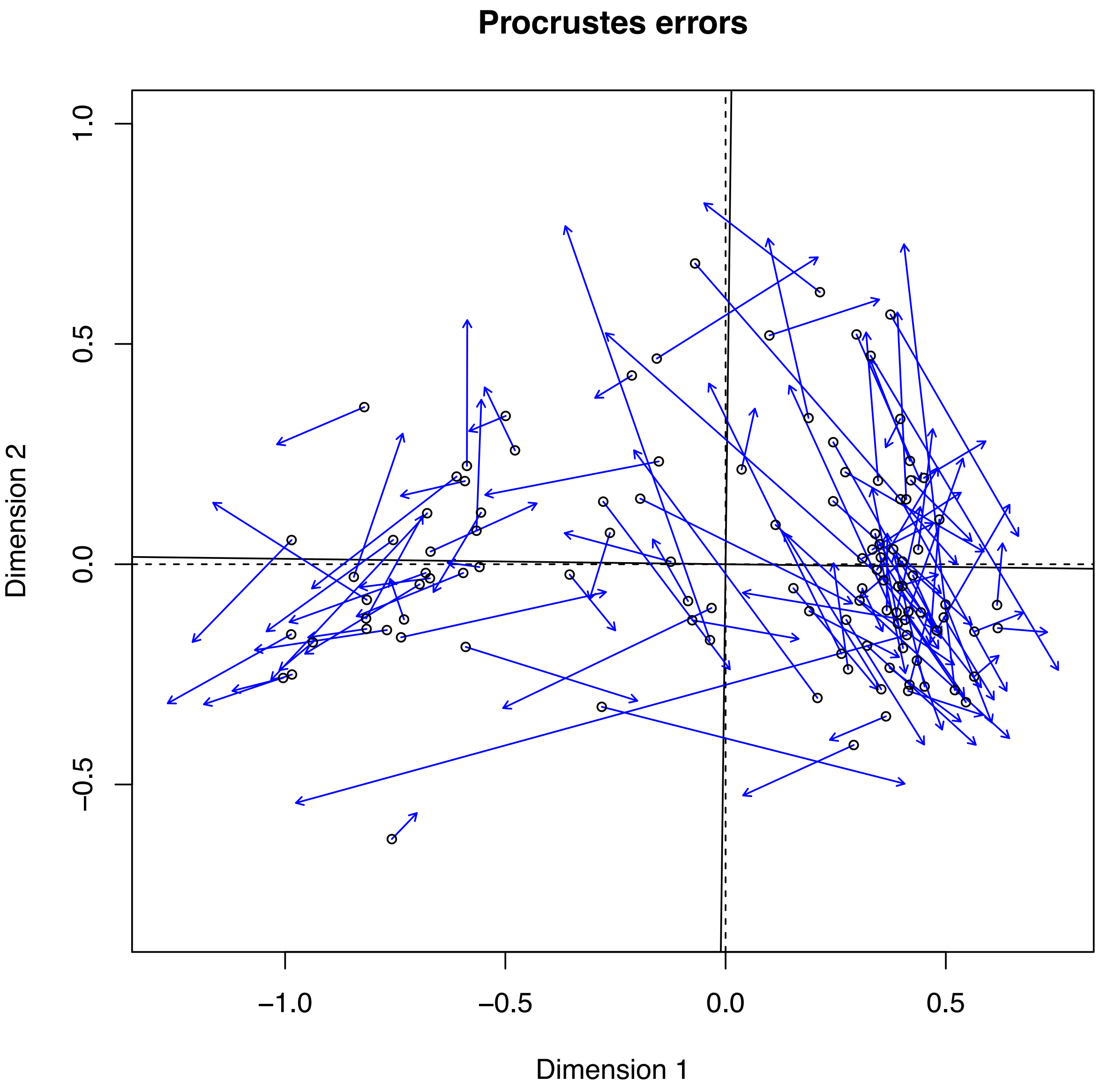

Procrustes analysis performed in the 110 samples with the two time points available. The analysis shows high overall concordance between the non-metric dimensional scaling (NMDS) matrices of the two datasets, eventhough a few individuals showed marked variations in microbiota ordination. The NMDS matrices were constructed using Bray-Curtis distance.

PROTEST[4] Statistics:
Procrustes Sum of Squares (m^2): 0.3475
Correlation in a symmetric Procrustes rotation: 0.8078
Significance: 0.001

Permutation: free
Number of permutations: 999

---

[4] *Jackson, D. A. Protest - A Procrustean Randomization Test of Community Environment Concordance. Ecoscience 2, 297–303 (1995).*

Supplementary figure 10. Composition of the fecal microbiota in the Stockholm dataset, according to *Bacteroides* vs. *Prevotella* cluster, HIV-1 status and HIV transmission group

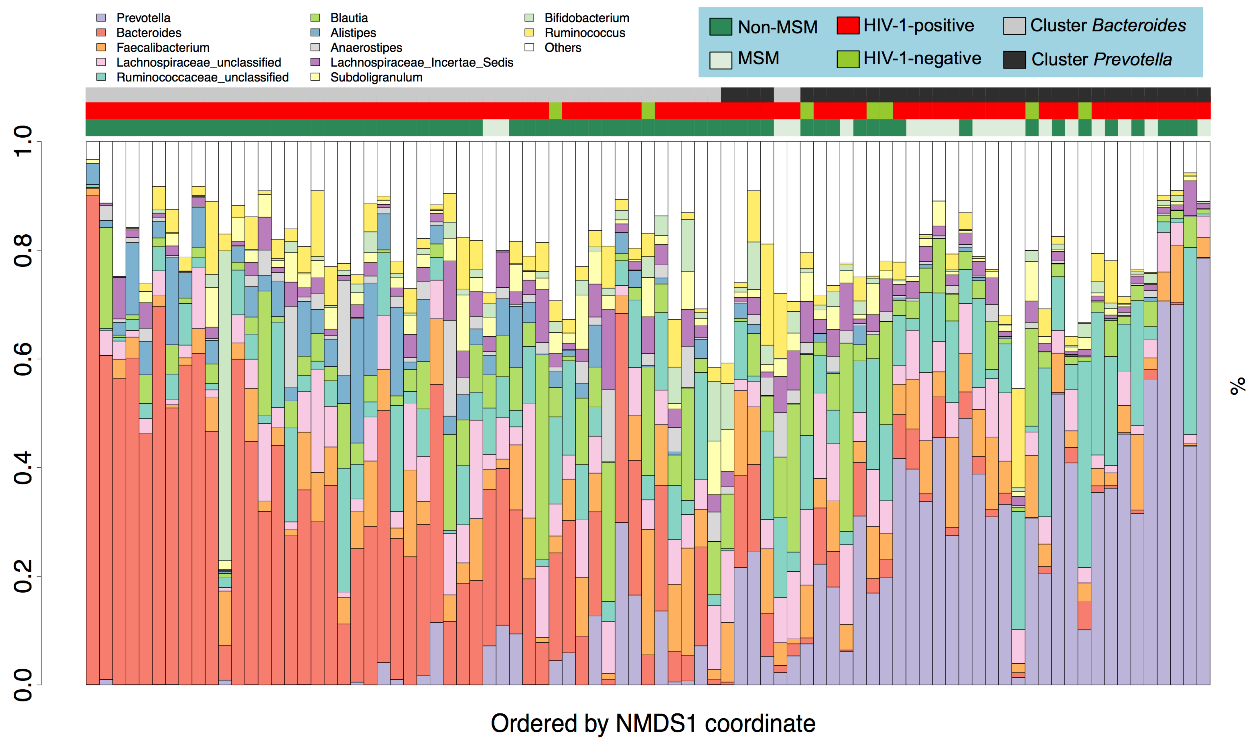

As shown in Figure 1 in the BCN0 dataset, the bacterial genus composition of the fecal microbiota in the Stockholm dataset also was largely linked to HIV transmission group, with MSM being enriched in the *Prevotella* cluster and non-MSM in the *Bacteroides* cluster. Genus with mean abundance of at least 2% across all samples are represented in colors; those with <2% abundance are grouped into the category "Others"

Supplementary figure 11A. Genus abundance plots by risk group: Barcelona test dataset (BCN0)

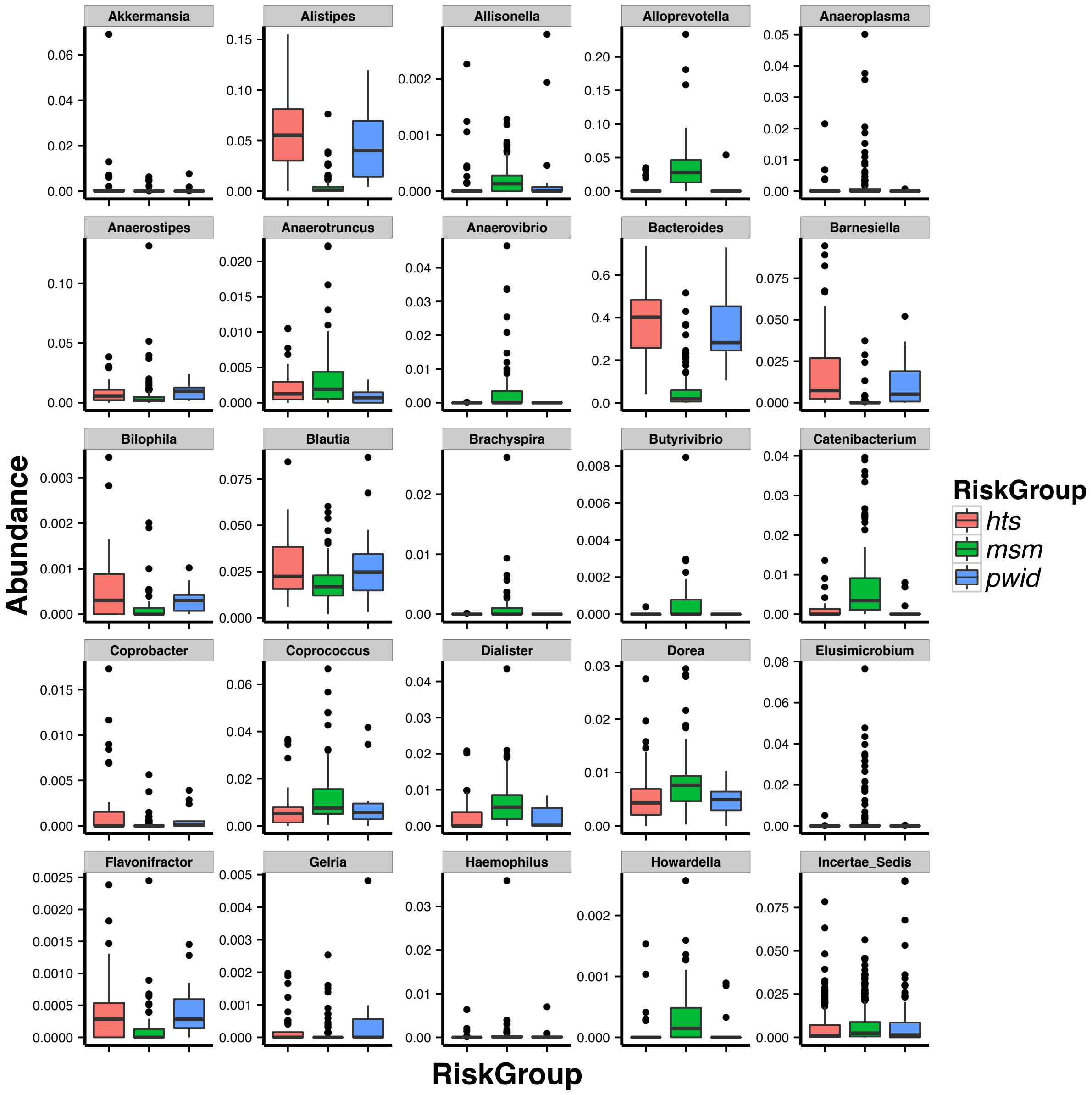

Only genus showing a Benjamini-Hochberg-corrected p-value <0.05 in a Kruskal-Wallis test are shown. One observes 2 seemingly mutually excluding patterns: some genus (like *Alloprevotella* or *Catenibacterium*, for example) are increased in MSM and decreased in HTS and PWID, whereas others (like *Bacteroides or Barnesiella*, for example) show the opposite trend.

Supplementary figure 11B. Genus abundance plots by risk group: Barcelona test dataset (BCN0)

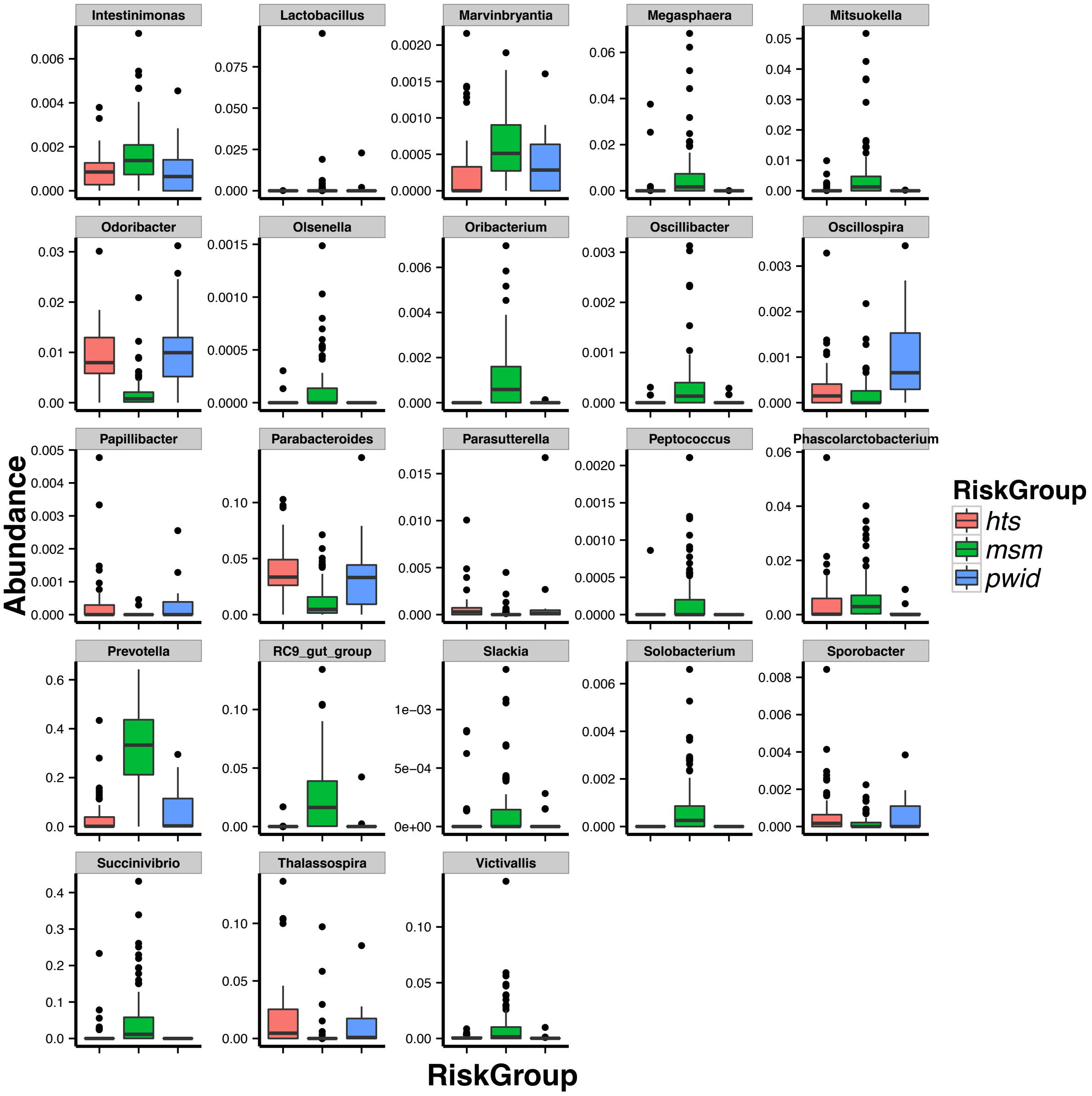

(continues from supplementary figure 10A) Only genus showing a Benjamini-Hochberg-corrected p-value <0.05 in a Kruskal-Wallis test are shown. One observes 2 seemingly mutually excluding patterns: some genus (like *Prevotella or Oribacterium*, for example) are increased in MSM and decreased in HTS and PWID, whereas others (like *Odoribacter* or *Thalassospira*, for example) show the opposite trend.

Supplementary figure 12A. Genus abundance plots by risk group: Barcelona internal validation dataset (BCN1)

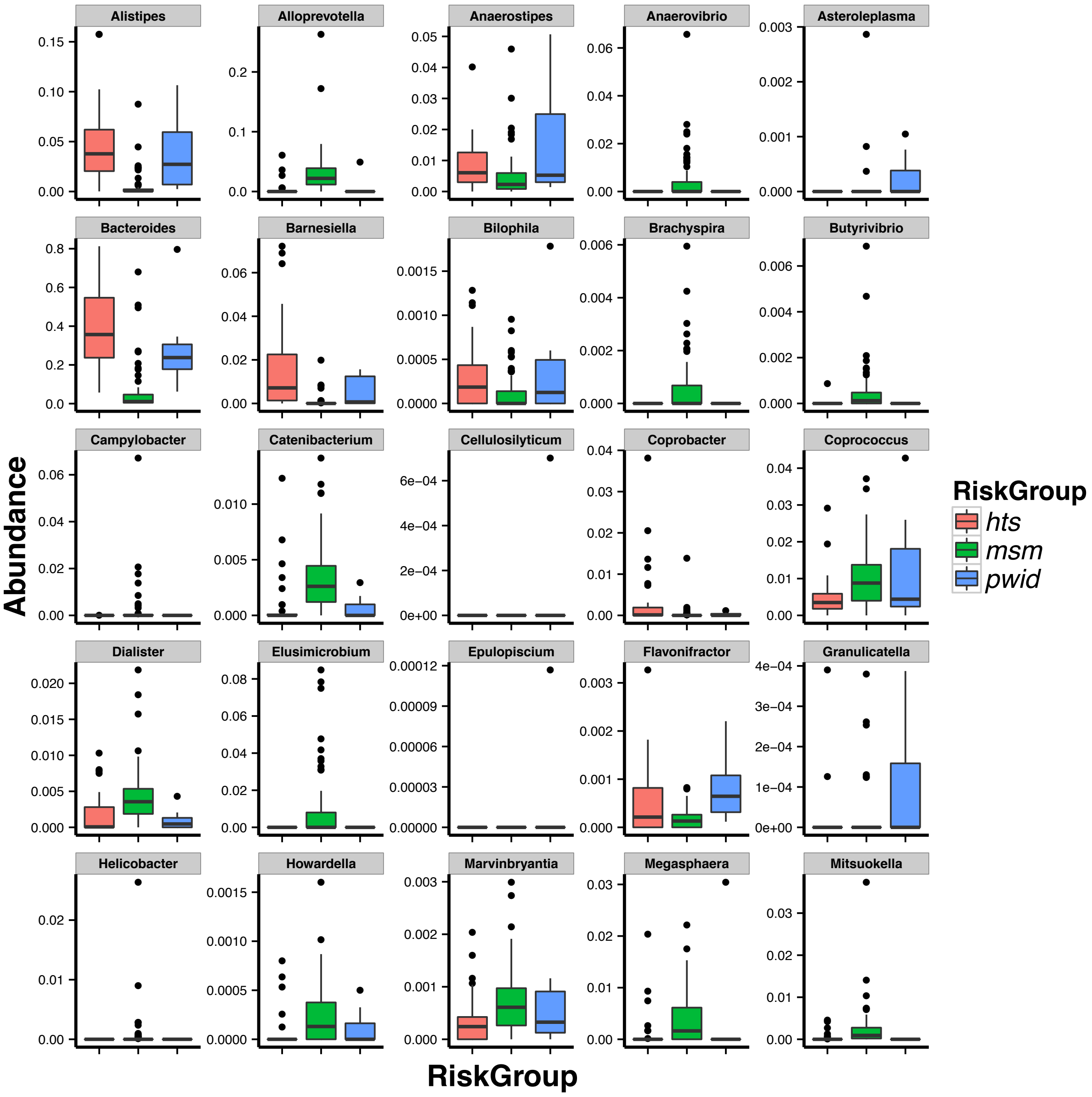

Only genus showing a Benjamini-Hochberg-corrected p-value <0.05 in a Kruskal-Wallis test are shown. One observes 2 seemingly mutually excluding patterns: some genus (like *Alloprevotella*, or *Catenibacterium*, for example) are increased in MSM and decreased in HTS and PWID, whereas others (like *Bacteroides or Barnesiella*, for example) show the opposite trend. Results match those from the Barcelona test (BCN1) and Stockholm external validation (STK) datasets.

Supplementary figure 12B. Genus abundance plots by risk group: Barcelona internal validation dataset (BCN1)

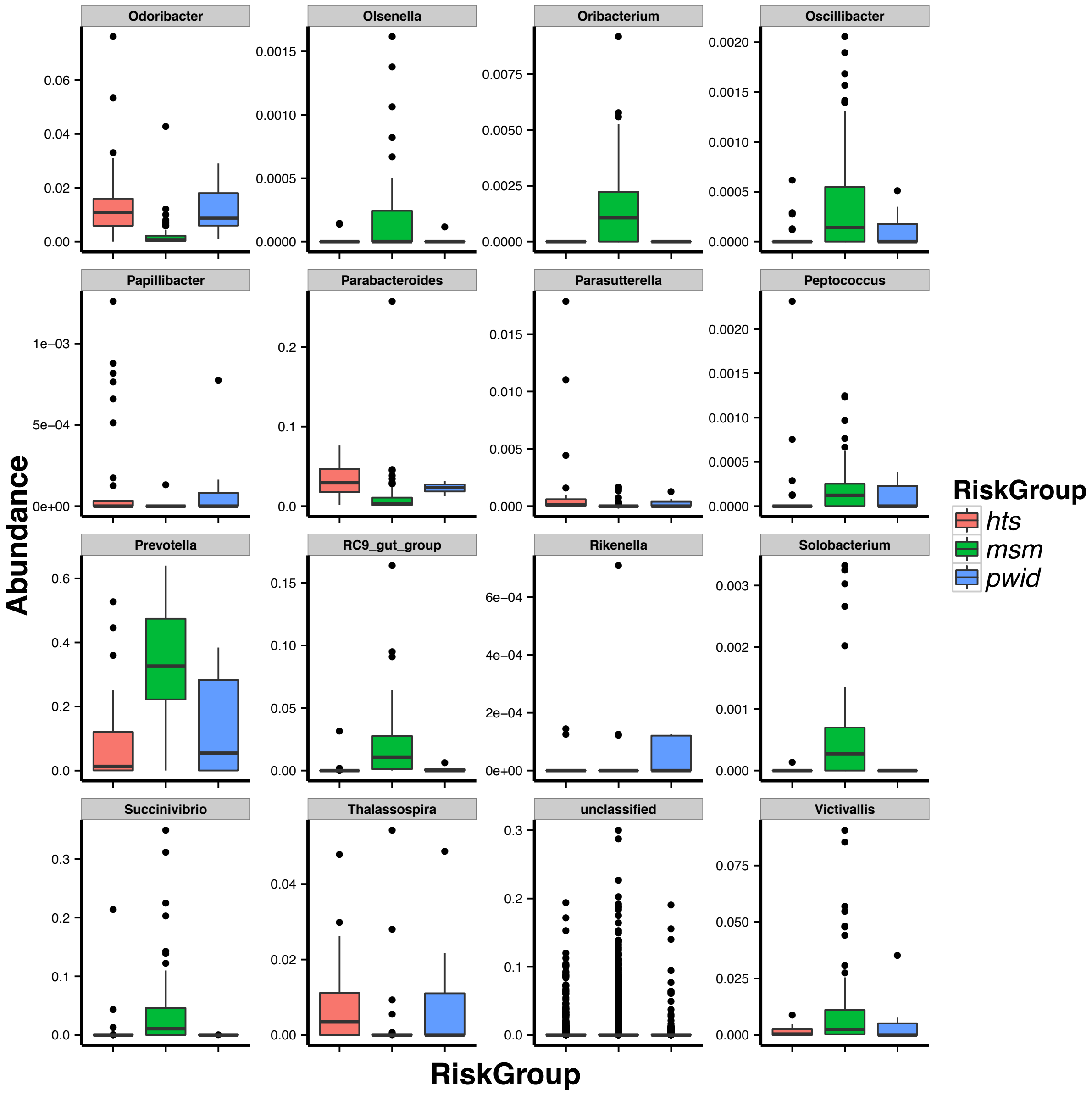

(continues from supplementary figure 11A) Only genus showing a Benjamini-Hochberg-corrected p-value <0.05 in a Kruskal-Wallis test are shown. One observes 2 seemingly mutually excluding patterns: some genus (like *Prevotella or Oribacterium*, for example) are increased in MSM and decreased in HTS and PWID, whereas others (like *Odoribacter* or *Thalassospira*, for example) show the opposite trend. Results match those from the Barcelona test (BCN0) and Stockholm external validation (STK) datasets.

Supplementary figure 13. Genus abundance plots by risk group: Stockholm external validation dataset (STK)

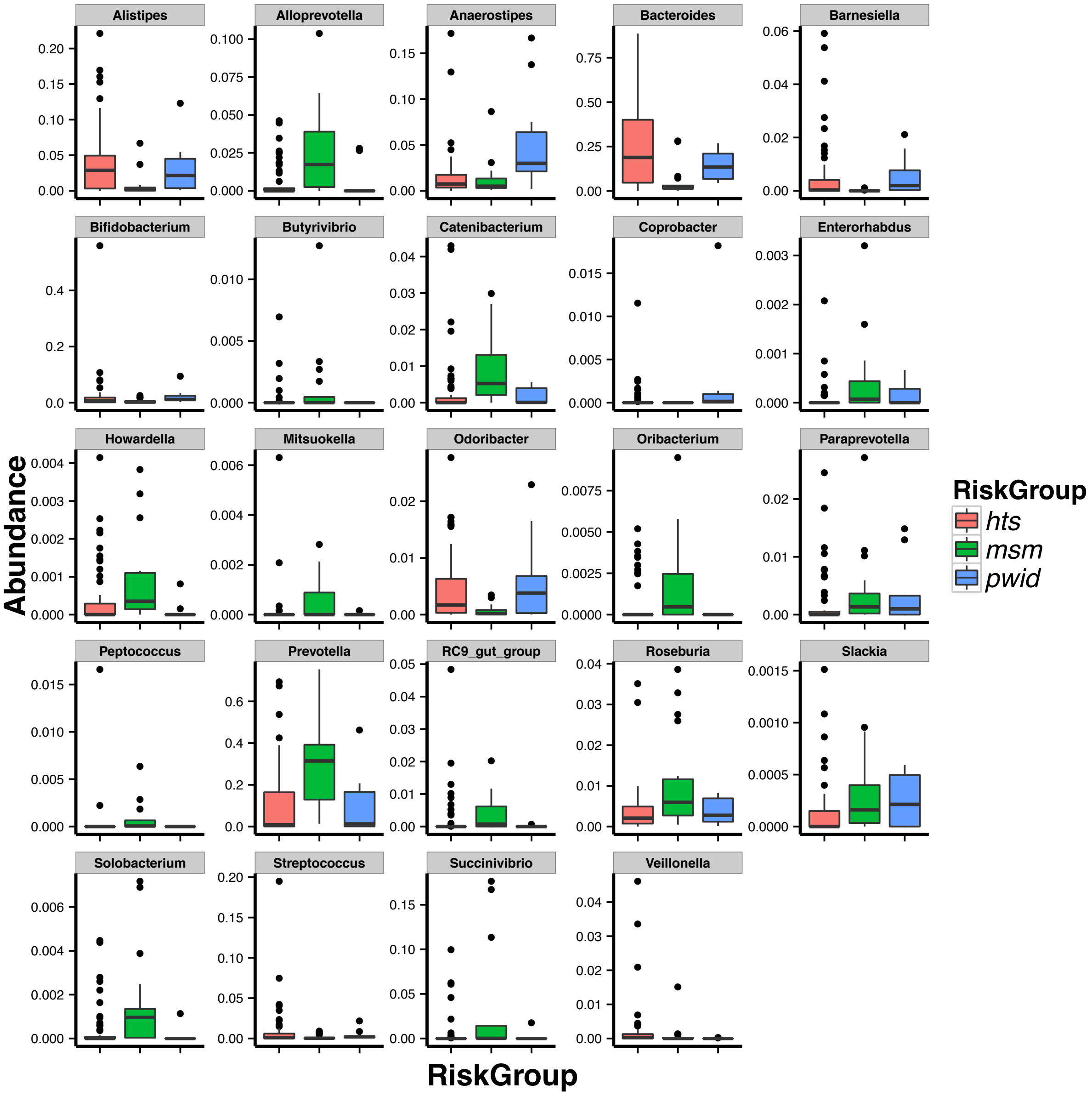

Only genus showing a Benjamini-Hochberg-corrected p-value <0.05 in a Kruskal-Wallis test are shown. One observes 2 seemingly mutually excluding patterns: some genus (like *Prevotella or Oribacterium*, for example) are increased in MSM and decreased in HTS and PWID, whereas others (like Bacteroides or *Odoribacter*, for example) show the opposite trend. Results match those from the Barcelona test (BCN0) and internal validation (BCN1) datasets.

Supplementary figure 14. Confirmation of the main study findings using LotuS[5], an independent sequence analysis pipeline

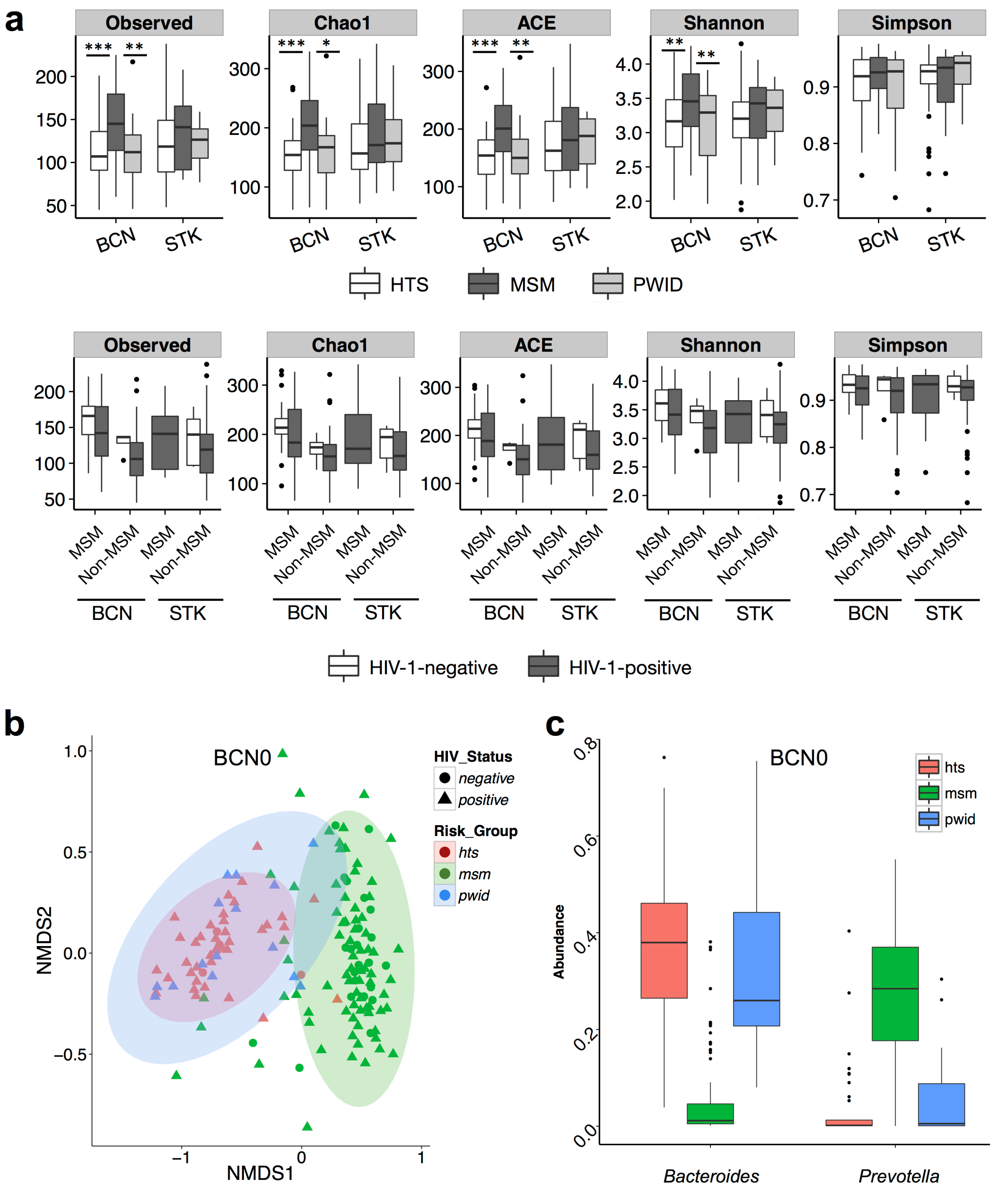

a, alpha diversity measures were equivalent to Figure 1, confirming that both HIV transmission group and HIV-1 infection modify the human fecal microbiome richness and diversity. **b, c,** beta diversity and genus abundance measures in the BCN0 dataset were equivalent to those of figure 2, confirming that the bacterial composition of the human fecal microbiome at the genus level is mainly linked to HIV transmission group. Other analyses, not shown here for simplicity, also replicated the results of the initial analysis approach.

---

[5] *Falk Hildebrand, Raul Tadeo, Anita Yvonne Voigt, Peer Bork and Jeroen Raes. LotuS: an efficient and user-friendly OTU processing pipeline. Microbiome 2014, 2:30 doi:10.1186/2049-2618-2-30.*

Supplementary figure 15. Fecal microbiota disbiosis by sexual preference group and HIV-1 status using LEFSe[6]

Barcelona (BCN0) Stockholm (STK)

by sexual preference

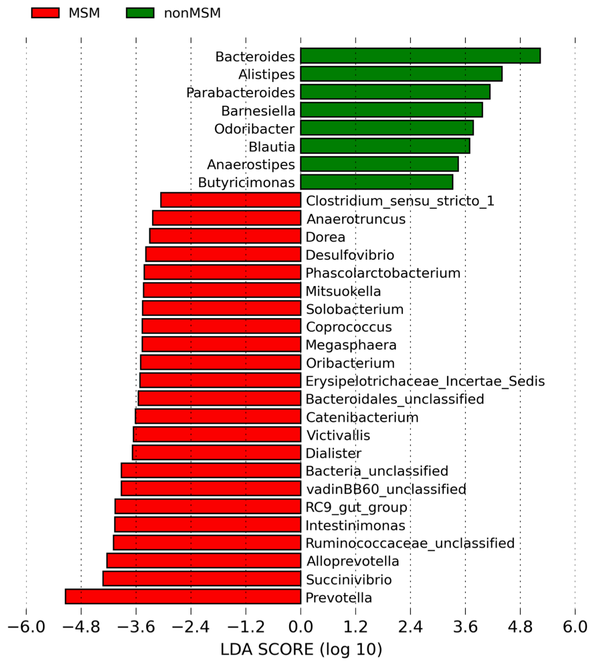

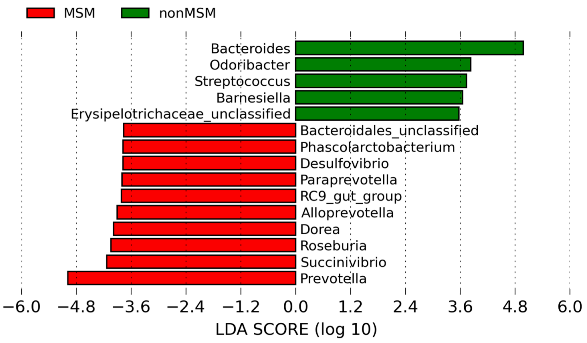

by HIV-1 status

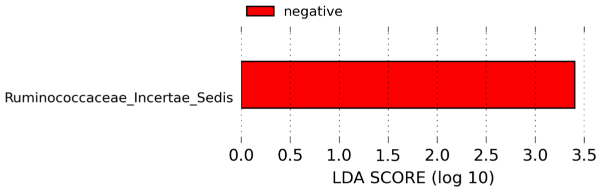

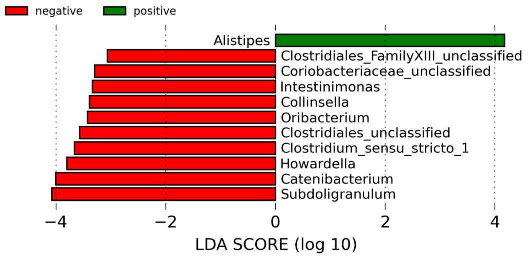

Bacterial genus enriched in MSM vs. Non-MSM individuals, regardless of HIV-1 status (class: HIV transmission group, subclass: HIV-1 status) (upper graphs), and in HIV-1-positive vs. HIV-1 negative subjects, regardless of HIV transmission group (class: HIV-1 status, subclass: HIV transmission group) (lower graphs) in Barcelona and Stockholm. Comparisons were done using the more stringent LEFSe criterion, i.e.: significant taxa had to differ between every pair of class values.

---

6 *Segata, N. et al. Metagenomic biomarker discovery and explanation. Genome Biol. 12, R60 (2011)*

Supplementary Figure 16. Lack of confounding of LASSO models by ethnicity, place of residence and stool consistency

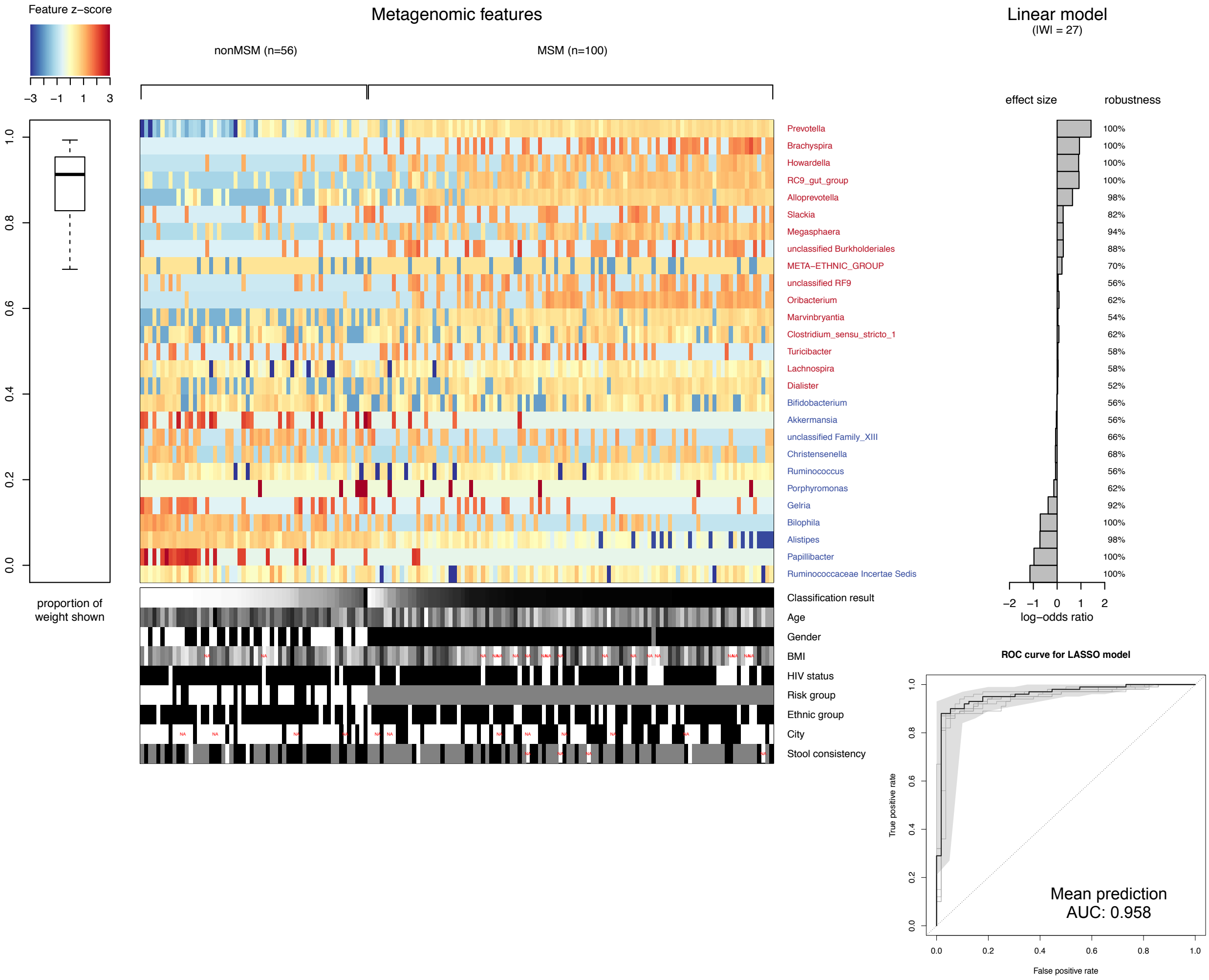

The LASSO models shown in Figure 3 were re-run including other metadata that could potentially confound the association between sexual orientation and gut microbiota composition, i.e., ethnic group, place of residence (city) and stool consistency. In the MSM model shown above, only ethnicity was picked up by the model, but with a low weight (see the META_ETHNIC_GROUP row in the model plot) and without a major change in model accuracy (see ROC curve, AUC 0.958). In the HIV model, not shown, none of these meta-variables was selected. Thereby, it is unlikely that ethnic group, place of residency and stool consistency might confound the LASSO models.

Supplementary Figure 17. Linear regression analysis of the association between normalized amounts of nutrients and microbiota composition in BCN0 samples

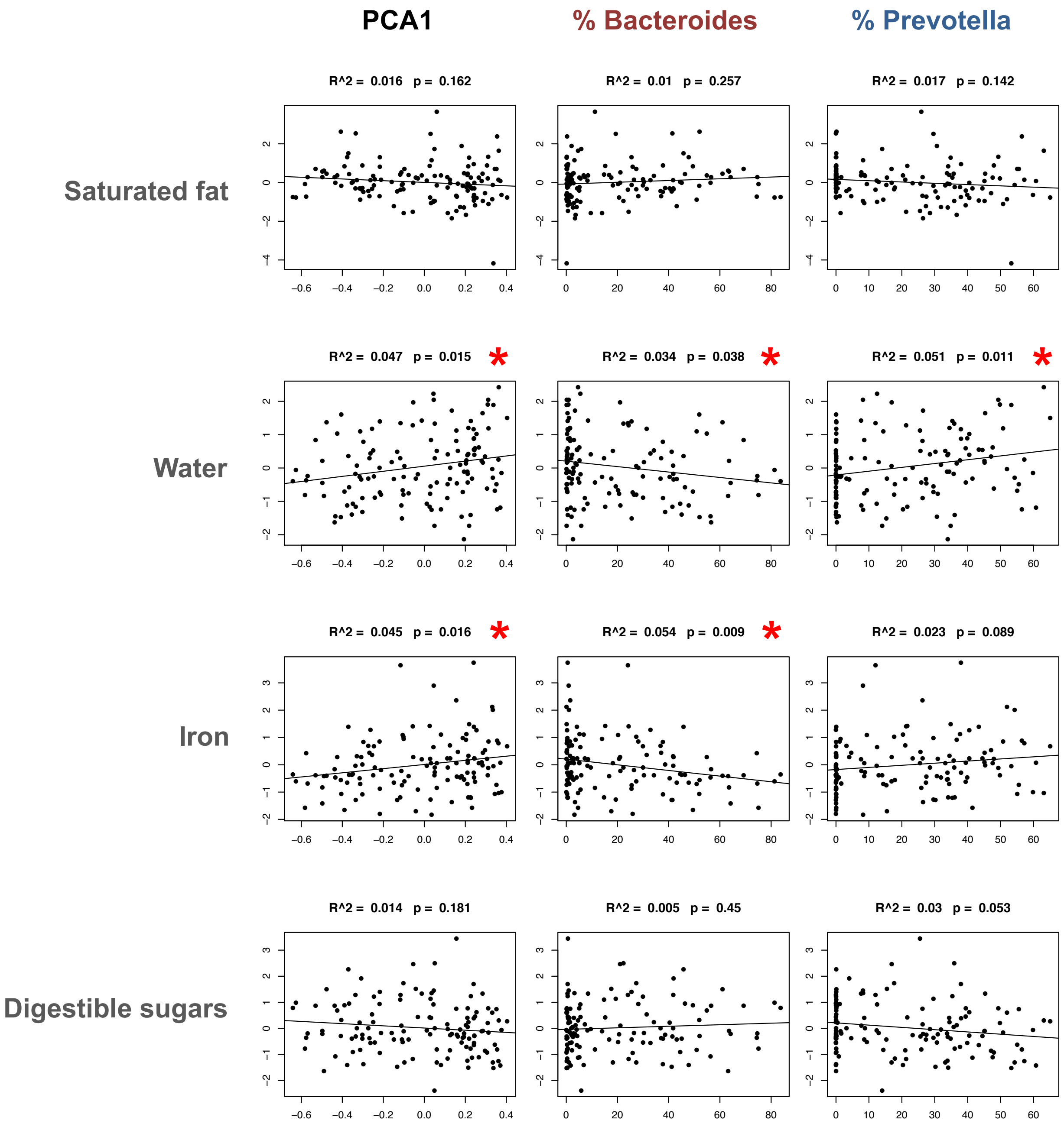

Nutrients chosen for this comparison were those showing strongest association signals in the Dirichlet multinomial regression model. The PCA1 axis captures 70% of variability of the microbiota composition. The axis moves, from left-to-right, from *Bacteroides* to *Prevotella* predominance. The percentage of the genus *Bacteroides* and *Prevotella* are shown in the X-axis in the corresponding figures. Y-axes show the amount of scaled residuals of energy-normalised nutrients. R-square and uncorrected p-values are shown. Red asterisks highlight correlations that retained statistical significance after correcting for multiple comparisons using a Benjamini-Hochberg FDR<0.1.

Supplementary Figure 18. Soluble markers of enterocyte damage, bacterial translocation and systemic inflammation

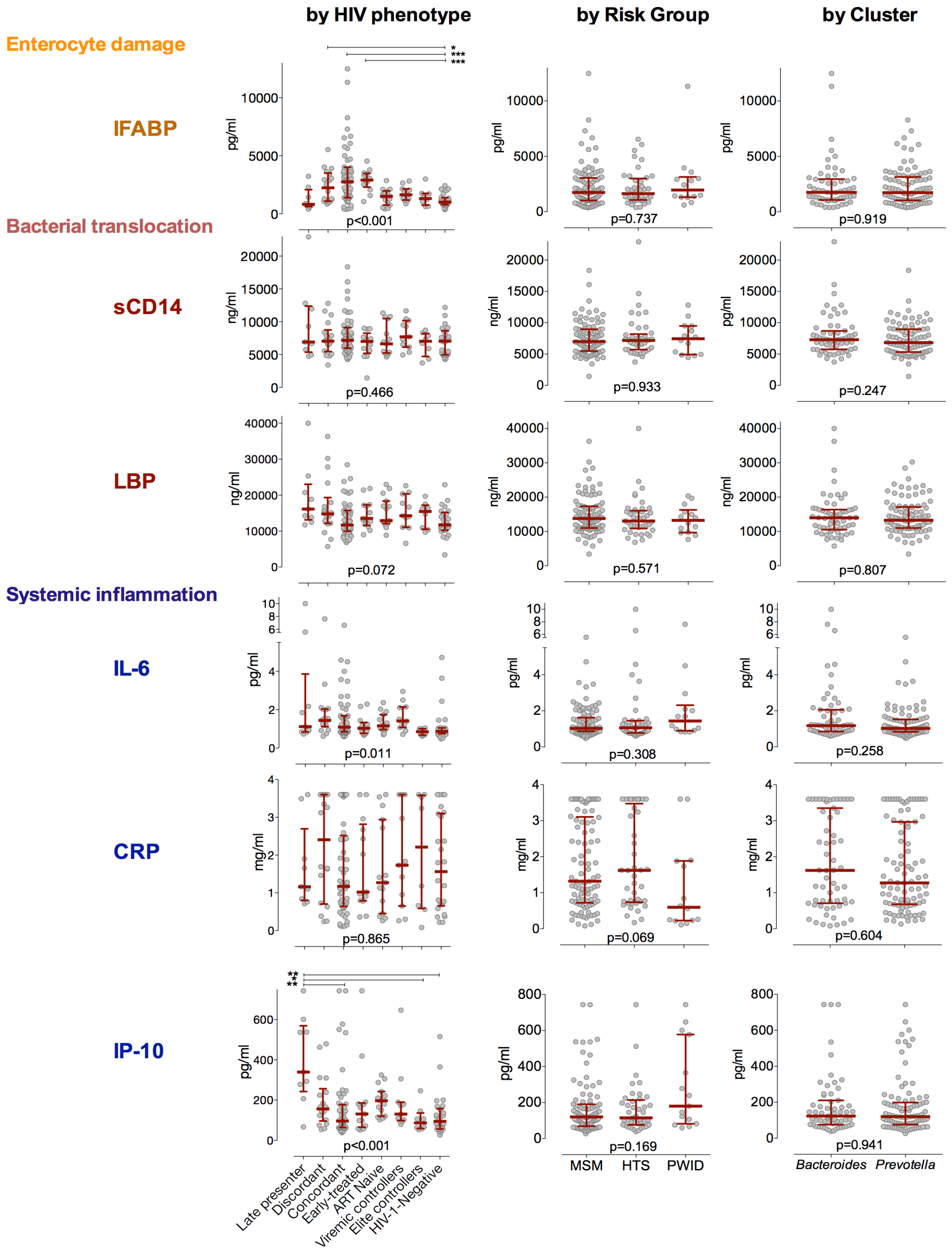

Whereas soluble markers showed expected associations with the different HIV-1 phenotypes, there were no differences by either risk group or by fecal microbiome cluster. Comparisons by phenotype and risk group were done using a Kruskal-Wallis test (global p-values at the bottom of each plot) using post-hoc tests when needed. Comparisons between clusters were done using a Mann-Whitney U test (p-values at the bottom of each plot). * p<0.1, ** p<0,05, *** p<0.001. IFABP, Intestinal Fatty Acid Binding Protein; sCD14, soluble CD14; LBP, lipopolysaccharide binding protein, IL-6, Interleukin-6; CRP, C-reactive protein; IP-10, interferon-gamma-inducible protein 10.

Supplementary Figure 19. Linear regression analysis of the association between each of the most abundant genus in the microbiota and soluble markers of intestinal integrity, bacterial translocation and systemic inflammation.

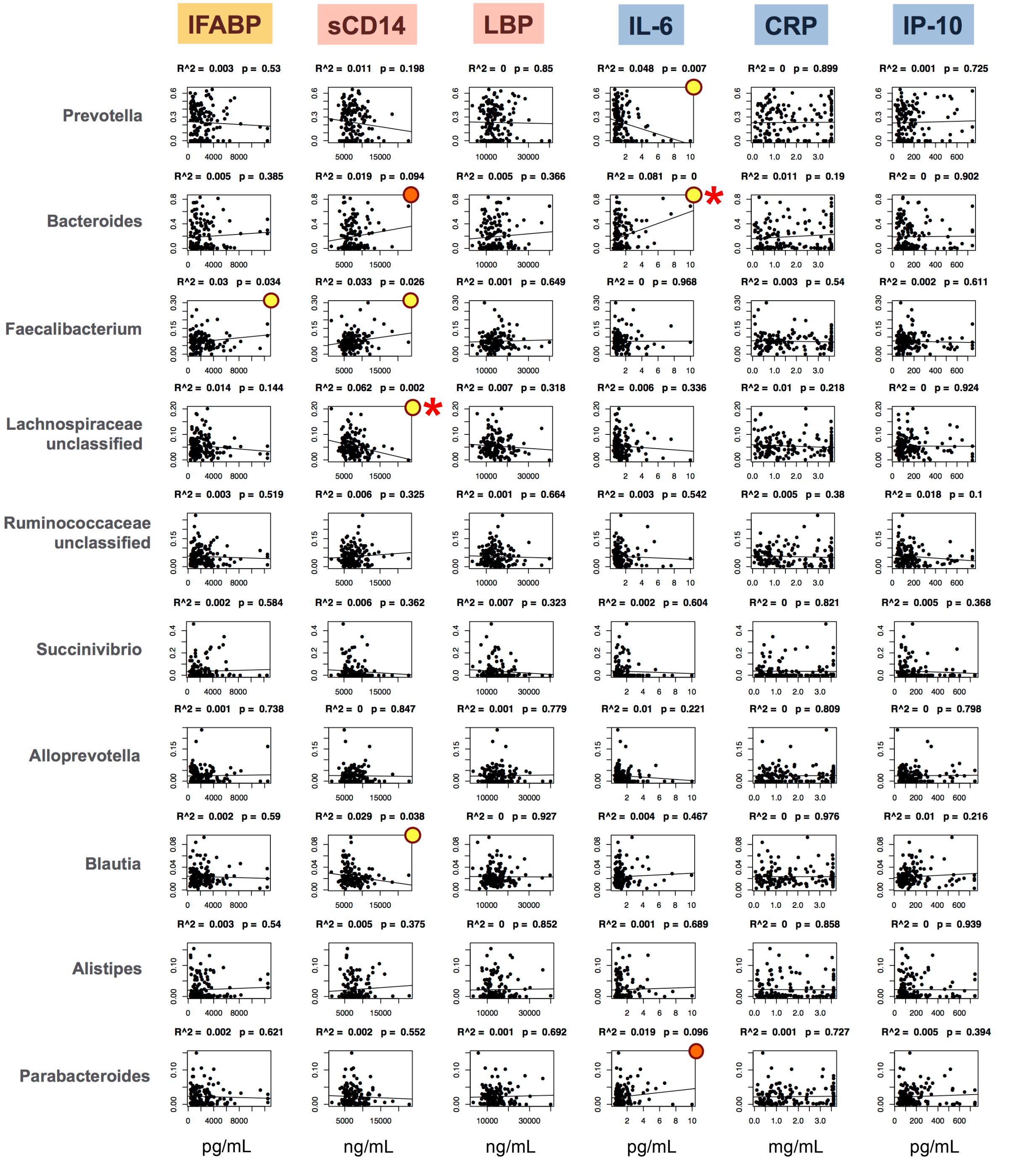

Genus selected had at least 2% mean abundance across the sample population. Y-axes show the relative amount of each genus (frequency) in the microbiota. X-axes show the amount of each soluble marker. Units are shown at the bottom of the figure. R-square and uncorrected p-values are shown. IFABP, Intestinal Fatty Acid Binding Protein; sCD14, soluble CD14; LBP, lipopolysaccharide binding protein, IL-6, Interleukin-6; CRP, C-reactive protein; IP-10, interferon-gamma-inducible protein 10.

Orange circle: Uncorrected P<0.1

Yellow circle: Uncorrected P<0.05

* Significant (P<0.1) after Benjamini-Hochberg correction
